# Temperature-Dependent Chirality in Halide Perovskites

**DOI:** 10.1021/acs.jpclett.4c01629

**Published:** 2024-07-31

**Authors:** Mike Pols, Geert Brocks, Sofía Calero, Shuxia Tao

**Affiliations:** † Materials Simulation & Modelling, Department of Applied Physics and Science Education, Eindhoven University of Technology, 5600 MB Eindhoven, Netherlands; ‡ Computational Chemical Physics, Faculty of Science and Technology and MESA+ Institute for Nanotechnology, University of Twente, 7500 AE Enschede, Netherlands

## Abstract

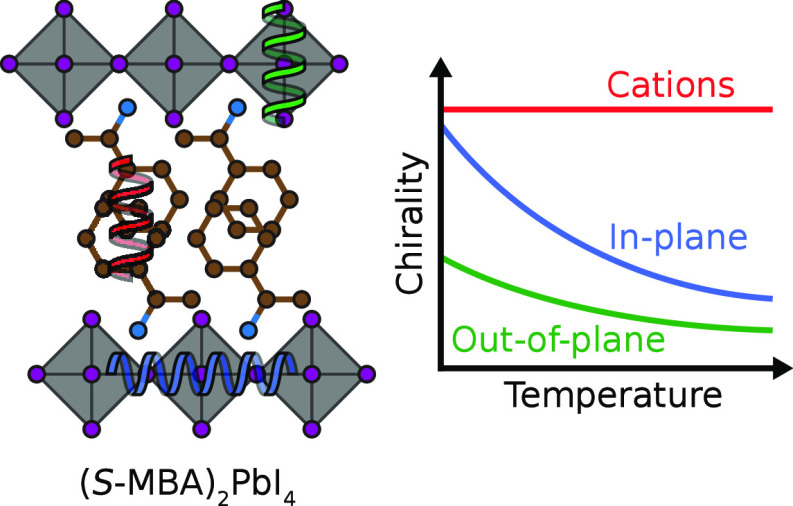

With the use of chiral organic cations in two-dimensional metal halide perovskites, chirality can be induced in the metal halide layers, which results in semiconductors with intriguing chiral optical and spin-selective transport properties. The chiral properties strongly depend upon the temperature, despite the basic crystal symmetry not changing fundamentally. We identify a set of descriptors that characterize the chirality of metal halide perovskites, such as MBA_2_PbI_4_, and study their temperature dependence using molecular dynamics simulations with on-the-fly machine-learning force fields obtained from density functional theory calculations. We find that, whereas the arrangement of organic cations remains chiral upon increasing the temperature, the inorganic framework loses this property more rapidly. We ascribe this to the breaking of hydrogen bonds that link the organic with the inorganic substructures, which leads to a loss of chirality transfer.

In materials science, chirality refers to a structural asymmetry where two mirror images of a molecule or crystal structure cannot be superimposed through any combination of translations and rotations. The mirror images of a crystal structure can commonly be connected via a mirror plane, inversion center, or improper rotation axis. The absence of such symmetry elements in a crystal signifies a chiral space group, for which the mirror images or enantiomers can be distinguished.^1^ A broad range of interesting properties emerge from chiral materials, including asymmetric chemical synthesis,^2^ circular dichroism (CD),^3^ circularly polarized photoluminescence (CPPL),^4^ chirality-induced spin selectivity (CISS),^5^ particular forms of second-harmonic generation,^6^ ferroelectricity,^7^ and topological quantum properties.^8^ It has resulted in wide interest in chiral materials over a broad range of domains in science.

With a focus on optoelectronic applications, chirality has been introduced in metal halide perovskites. Metal halide perovskites in particular exhibit excellent optoelectronic properties with a high tunability, making them promising candidates for solar cells,^9^ light-emitting diodes (LEDs),^10^ and photodetectors.^11^ The perovskite crystal lattice is a framework composed of vertex-linked inorganic MX_6_ octahedra (M = metal and X = halide), intercalated with inorganic or organic cations.^12^ Small cations result in the formation of three-dimensional (3D) MX_6_ frameworks, whereas larger organic cations facilitate the growth of lower dimensional frameworks; two-dimensional (2D), one-dimensional (1D), or zero-dimensional (0D).^13,14^ The resulting materials, although 3D crystals, are commonly termed 2D, 1D, or 0D perovskites.

The flexibility of the perovskite crystal structure paves the way for the incorporation of chirality in halide perovskites using large chiral organic ligands.^15,16^ Lead and tin halide 2D perovskites currently attract most attention, and optical phenomena that are typical of chiral materials have been demonstrated in these materials, such as CD^17−23^ and CPPL.^18−20,23,24^ Concerning electronic transport, these materials also show a high degree of CISS.^21,25^ The optoelectronic properties that draw most interest typically stem from chirality present in the MX_6_ framework, because the electronic states around the band gap, which determine electronic transport and the onset of the optical transitions in these perovskites, originate from that framework. Because the latter does not inherently contain any chiral structural units, it requires a chiral structural distortion of this framework originating from the organic ligands.

By comparison of various sets of chiral 2D perovskites, several studies have elucidated the relationship between structural chirality and the magnitude of spin splitting in the electronic band structure^26,27^ as well as chiral optical activity.^28−30^ Although the fundamental mechanisms underlying this chiral optical activity and spin-selective transport are not well-understood, these investigations have identified some distinct structural features that can be related to these processes. Specifically, a structural asymmetry in the tilting of the metal halide octahedra within the inorganic framework^26,27,30^ and the hydrogen bonding at the interface between organic cations and the inorganic framework^28,29^ have been highlighted. Another significant experimental observation is the pronounced temperature dependence of the chiral optical properties of chiral perovskites,^19,24^ suggesting a decrease of chirality at elevated temperatures. To enhance the understanding of the temperature dependence of the chiral properties, it is essential to identify the chiral elements within the overall structure as well as the interplay between them and understand their evolution at finite temperatures.

In this letter, we introduce a comprehensive set of structural descriptors that characterize chirality in 2D halide perovskite structures as a whole. Different descriptors capture the chirality in the arrangement of organic cations and the in-plane and out-of-plane chirality of the 2D MX_6_ framework, as illustrated for a selection of 2D lead halide perovskites. We study the temperature dependence of these structural chirality descriptors through molecular dynamics (MD) simulations with on-the-fly machine-learning force fields (MLFFs) based on density functional theory (DFT) calculations, which we apply to the archetype chiral 2D perovskite MBA_2_PbI_4_. These simulations indicate that chirality in the organic cation arrangement persists up to a relatively high temperature, whereas the chirality in the lead halide planes is diminished substantially. The cause of this is found in the NH_3_^+^ groups that link the organic cations to the lead halide framework, whose rotations at elevated temperatures inhibit chirality transfer from the cation to the framework.

To understand the transfer mechanism of structural chirality in halide perovskites, we begin by outlining the geometrical features that we use to capture the symmetry breaking in the crystal structure of the archetype 2D chiral perovskite with its two enantiopure crystals (*S*-MBA)_2_PbI_4_ and (*R*-MBA)_2_PbI_4_. Here, *S*-MBA^+^ and *R*-MBA^+^ indicate the two enantiomers of the methylbenzylammonium cation (MBA^+^). This crystal structure, shown in Figure 1a, is composed of layers of corner-sharing metal halide (PbI_6_) octahedra, between which the *S*-MBA^+^ and *R*-MBA^+^ cations are located. The compound crystallizes in the *P*2_1_2_1_2_1_ space group,^26^ which has a 2_1_ screw axis in the direction of all lattice vectors, lacks an inversion center, and is thus chiral. In contrast, a racemic mixture of *S*-MBA^+^ and *R*-MBA^+^ is found to crystallize in a (*rac*-MBA)_2_PbI_4_ structure with a *P*2_1_/*c* space group,^20^ which, besides its 2_1_ screw axis, also has an inversion center and is therefore achiral.

**Figure 1:**
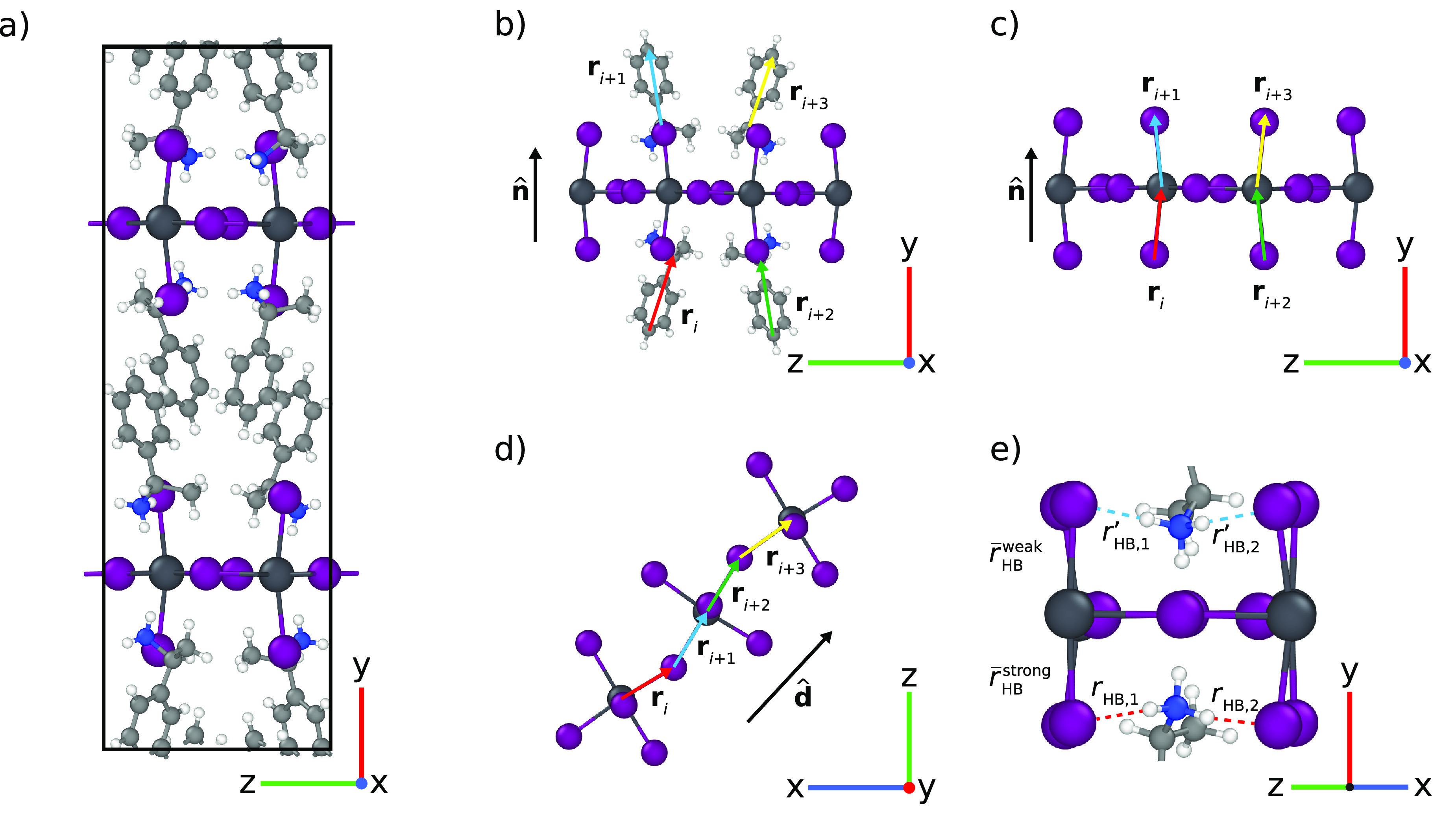
Overview of structural descriptors for 2D metal halide perovskites. (a) Unit cell of the chiral (*S*-MBA)_2_PbI_4_ perovskite. (b) Chirality of organic cations (*ϵ*_A_2__). (c) Out-of-plane chirality of the inorganic framework (*ϵ*_MX_4__^⊥^). (d) In-plane chirality of the inorganic framework (*ϵ*_MX_4__^∥^). (e) Hydrogen bond asymmetry (Δ*r*_HB_). The various structural vectors used in the vector pairs and projection directions are shown in the panels.

We employ a set of structural descriptors to probe the extent to which symmetries are broken in various parts of the crystal structures. The descriptors assess the chirality of the arrangement of the organic cations (Figure 1b), the out-of-plane and in-plane chirality of the inorganic framework (panels c and d of Figure 1), and the asymmetry in the hydrogen bonding (Figure 1e). We find that these descriptors are more useful than others for characterizing structural chirality. Other possible descriptors, which measure distortions within and between the inorganic octahedra, are listed in Note 1 of the Supporting Information.

In characterizing the chirality of various components of chiral perovskites, we used the concept of vector chirality. For magnetic systems, this concept has been used to assign a chirality or handedness to spin configurations.^31−33^ Adapting the vector chirality concept from spin systems to crystal structures, we replace the spin orientations with the orientation vectors of the chemical bonds. As such, we define the structural vector chirality **ϵ** as the sum over pairwise cross products of neighboring orientation vectors as
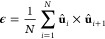
1where **û**_*i*_ = **r**_*i*_/|**r**_*i*_| is the normalized bond orientation vector and *N* is the number of vectors within a selected set; see for example panels b–d of Figure 1. To measure the handedness in a specific direction **p̂**, we project the vector chirality **ϵ** along that direction as
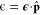
2to obtain the scalar structural chirality *ϵ*. A non-zero value of *ϵ* indicates a net handedness, with the sign making a distinction between left and right, whereas a value of zero implies a lack of chirality in that particular component and the direction of the structure.

We assign a structural vector chirality to individual parts of the structure, the first of which pertains to the arrangement of the organic cations. As a first step, we assign a direction vector **r**_*i*_ to each organic cation; see Figure 1b. The exact definitions of these direction vectors for the various cations are shown in Note 2 of the Supporting Information. Subsequently, we used the direction vectors of four cations along the inorganic layers (Figure 1b) to calculate the structural vector chirality according to eq 1. Note that cations marked by **r**_*i*_ and **r**_*i*+3_ are related by an action along an in-plane 2_1_ screw axis, as are the molecules marked by **r**_*i*+1_ and **r**_*i*+2_. The resulting chirality vector **ϵ**_A_2__ is then projected according to eq 2, in the direction normal to the metal halide plane *ϵ*_A_2__ = **ϵ**_A_2__ · **n̂**.

To characterize the out-of-plane chirality of the inorganic framework, we use the M–X bonds perpendicular to the inorganic layers to define direction vectors and treat those in an analogous way to the direction vectors of the organic cations (Figure 1c). This gives the out-of-plane chirality vector **ϵ**_MX_4__^⊥^, which, projected normal to the inorganic planes, yields *ϵ*_MX_4__^⊥^ = **ϵ**_MX_4__^⊥^ · **n̂**. The chirality vector of the inorganic framework in plane (**ϵ**_MX_4__^∥^) is determined using the direction vectors of in-plane M–X bonds, selected along lines **d̂** that connect the inorganic octahedra; see Figure 1d. This chirality vector is subsequently projected along the direction of these lines as *ϵ*_MX_4__^∥^ = **ϵ**_MX_4__^∥^ · **d̂**. Projection in the **d̂** direction captures the in-plane chirality of the structure. Other possible projections are discussed in Note 4 of the Supporting Information.

To make a connection with previous work on chirality in metal halide perovskites,^26,28,29^ we also probe the symmetry breaking in the hydrogen bonds, which form the links between NH_3_ groups of the organic cations and halide ions in the inorganic framework. We quantify this asymmetry through the difference in the hydrogen bond lengths (i.e. N–H···X bonds) between the two opposing cations above and below the inorganic layer (Figure 1e). For each cation, we compute the average bond length of the two shortest hydrogen bonds *r̅*_HB_ = (*r*_HB,1_ + *r*_HB,2_)/2, where *r*_HB,1_ and *r*_HB,2_ refer to the shortest and second shortest hydrogen bonds. The hydrogen bond asymmetry Δ*r*_HB_ is subsequently determined for every inorganic cage as Δ*r*_HB_ = *r̅*_HB_^weak^ – *r̅*_HB_^strong^, with *r̅*_HB_^weak^ and *r̅*_HB_^strong^ as the average hydrogen bond length of the cation with the longest and shortest hydrogen bonds, respectively. A non-zero value of Δ*r*_HB_ implies a breaking of symmetry, whereas a value of zero indicates highly symmetric hydrogen bonding of the cations above and below the metal halide plane.

Next, we used the above-mentioned structural descriptors to assess the structural chirality of a set of 2D perovskites from experiments. We optimized the structures using DFT calculations with the SCAN exchange–correlation functional^34^ in the Vienna *Ab initio* Simulation Package (VASP).^35−37^ The full details of this structural optimization can be found in Note 3 of the Supporting Information. The resulting descriptor values are listed in Table 1. A more detailed overview of the structural descriptors in 2D perovskites is shown in Note 4 of the Supporting Information.

**Table 1:** Structural Descriptors Probing Structural Chirality and Bond Asymmetry in Chiral 2D Perovskite Structures

perovskite	reference	*ϵ*_A_2__ (×10^–3^)	*ϵ*_MX_4__^∥^ (×10^–3^)	*ϵ*_MX_4__^⊥^ (×10^–3^)	Δ*r*_HB_ (Å)
(*S*-MBA)_2_PbI_4_	(26)	+46.490	+4.534	+1.560	0.036
(*R*-1NEA)_2_PbBr_4_	(26)	+4.481	+12.629	–9.319	0.058
(*R*-2NEA)_2_PbBr_4_	(29)	–10.791	–4.394	–2.021	0.107
(*R*-4-Cl-MBA)_2_PbBr_4_	(27)	–21.903	+7.762	+0.229	0.031
(*S*-1-Me-HA)_2_PbI_4_	(27)	–13.333	+13.230	–0.648	0.033
(*S*-2-Me-BuA)_2_PbBr_4_	(27)	+38.809	–4.302	+0.849	0.000

Focusing on chiral (*S*-MBA)_2_PbI_4_, we find non-zero values for the chirality of the arrangement of cations (*ϵ*_A_2__ = +46.490 × 10^–3^), which through asymmetric hydrogen bonds (Δ*r*_HB_ = 0.036 Å) transfer the breaking of this structural symmetry to the inorganic framework (*ϵ*_MX_4__^∥^ = +4.534 × 10^–3^ and *ϵ*_MX_4__^⊥^ = +1.560 × 10^–3^). Notably, we find that the structural descriptors appropriately distinguish between the structural enantiomers of MBA_2_PbI_4_, as shown in Table S6 in Note 4 of the Supporting Information. For example, chiral (*R*-MBA)_2_PbI_4_, the structural enantiomer of (*S*-MBA)_2_PbI_4_, has identical descriptor values with an opposite sign, which indicates the opposite handedness of the two enantiomers. We note that we do not find an opposite sign for the hydrogen bond asymmetry, which stems from its definition that results in Δ*r*_HB_ ≥ 0 in static structures. Moreover, for achiral (*rac*-MBA)_2_PbI_4_, the structural descriptors have values of zero (*ϵ*_A_2__ = *ϵ*_MX_4__^⊥^ = *ϵ*_MX_4__^∥^ = 0 and Δ*r*_HB_ = 0.0 Å), which implies an absence of any structural chirality in these perovskites.

Shifting our focus from MBA_2_PbI_4_ to the other chiral 2D perovskites, we can assess the variance in the structural chirality across perovskite structures. As a result of the molecule-dependent definition of the cation orientation vectors (Note 2 of the Supporting Information), the chirality of the cation arrangements of widely different molecular species cannot be directly compared. In contrast, as a result of the similarity of the inorganic framework across compounds, the structural chirality of the inorganic framework, both in-plane and out-of-plane, can be compared across compounds. The comparison shows that the various components of the perovskite lattice can exhibit structural symmetry breaking to varying extents, independent of each other. For example, (*R*-1NEA)_2_PbBr_4_ has a high in-plane and out-of-plane framework chirality, whereas (*S*-1-Me-HA)_2_PbI_4_ only has a high in-plane chirality with a rather small out-of-plane chirality. Interestingly, the comparison shows that a larger asymmetry in the hydrogen bonds does not necessarily imply a larger structural distortion in the inorganic framework. However, all chiral 2D perovskites exhibit symmetry breaking in the various components of the crystal structure, as demonstrated by the non-zero structural descriptors. Finally, we do not observe a strong correlation between the structural chirality and spin splitting in 2D perovskites (Note 5 of the Supporting Information), indicating that there are potentially more effects relevant to spin splitting in addition to chiral symmetry breaking.

Having defined descriptors capable of identifying structural chirality in perovskite structures, we now focus on analyzing the effects that finite temperatures have on structural chirality. This analysis allows us to characterize the persistence of chirality as a function of the temperature, gaining insights into the differences in chirality between the various components in the perovskite lattice and the effectiveness of chirality transfer between those components. We will use the archetype 2D chiral perovskite, MBA_2_PbI_4_, as an example.

To assess the effects of the temperature, we use MLFFs to study the lattice dynamics of 2D perovskites with MD simulations. The MLFFs are trained against energies, forces, and stresses from DFT calculations, using the on-the-fly learning scheme to sample structures from first-principles MD simulations as described in refs (38 and 39). The DFT reference data were obtained using the SCAN exchange–correlation functional in calculations in VASP.^35−37^ The full details of the training procedure, the training sets, and the validation of the trained MLFFs are shown in Note 6 of the Supporting Information. We highlight that the trained MLFFs possess a high degree of transferability across perovskite structures. Specifically, the model trained on (*S*-MBA)_2_PbI_4_ describes both (*R*-MBA)_2_PbI_4_ and (*rac*-MBA)_2_PbI_4_ with high accuracy, as shown in Figures S5 and S7 of the Supporting Information.

Using the MLFFs, we subject the three variants of MBA^+^-based perovskites, (*S*-MBA)_2_PbI_4_, (*R*-MBA)_2_PbI_4_, and (*rac*-MBA)_2_PbI_4_, to a temperature of 50 K at atmospheric pressure in *NpT* MD simulations. As a result of thermal motion of the atoms within the structures, the four descriptors (*ϵ*_A_2__, *ϵ*_MX_4__^∥^, *ϵ*_MX_4__^⊥^, and Δ*r*_HB_) are no longer single-valued but form distributions around mean values. The latter values are close to the values reported for the static structures in Table 1, as shown in Figure 2. Focusing on the descriptors that differ in sign between (*S*-MBA)_2_PbI_4_ and (*R*-MBA)_2_PbI_4_ (panels a–c of Figure 2), we observe that, in some cases, the widths of the distributions as a result of thermal broadening are larger than the distance between the maxima of the peaks for (*S*-MBA)_2_PbI_4_ and (*R*-MBA)_2_PbI_4_. This is, for example, the case for the in-plane chirality (*ϵ*_MX_4__^∥^; Figure 2b) and, in particular, for the out-of-plane chirality (*ϵ*_MX_4__^⊥^; Figure 2c) of the inorganic framework. Especially for *ϵ*_MX_4__^⊥^, thermal motion makes it very difficult to distinguish between (*S*-MBA)_2_PbI_4_ and (*R*-MBA)_2_PbI_4_. In contrast, the descriptor for the chirality in the organic cation arrangement (*ϵ*_A_2__; Figure 2a) is only slightly affected by thermal motion. It can also be seen that the hydrogen bond asymmetry (Δ*r*_HB_; Figure 2d) is almost completely gone as a result of thermal motion. To support our findings, we also plot the descriptor distributions for (*rac*-MBA)_2_PbI_4_ (panels e–h of Figure 2), which are found to be symmetric around zero, indicating that this crystal structure remains achiral at a finite temperature.

**Figure 2:**
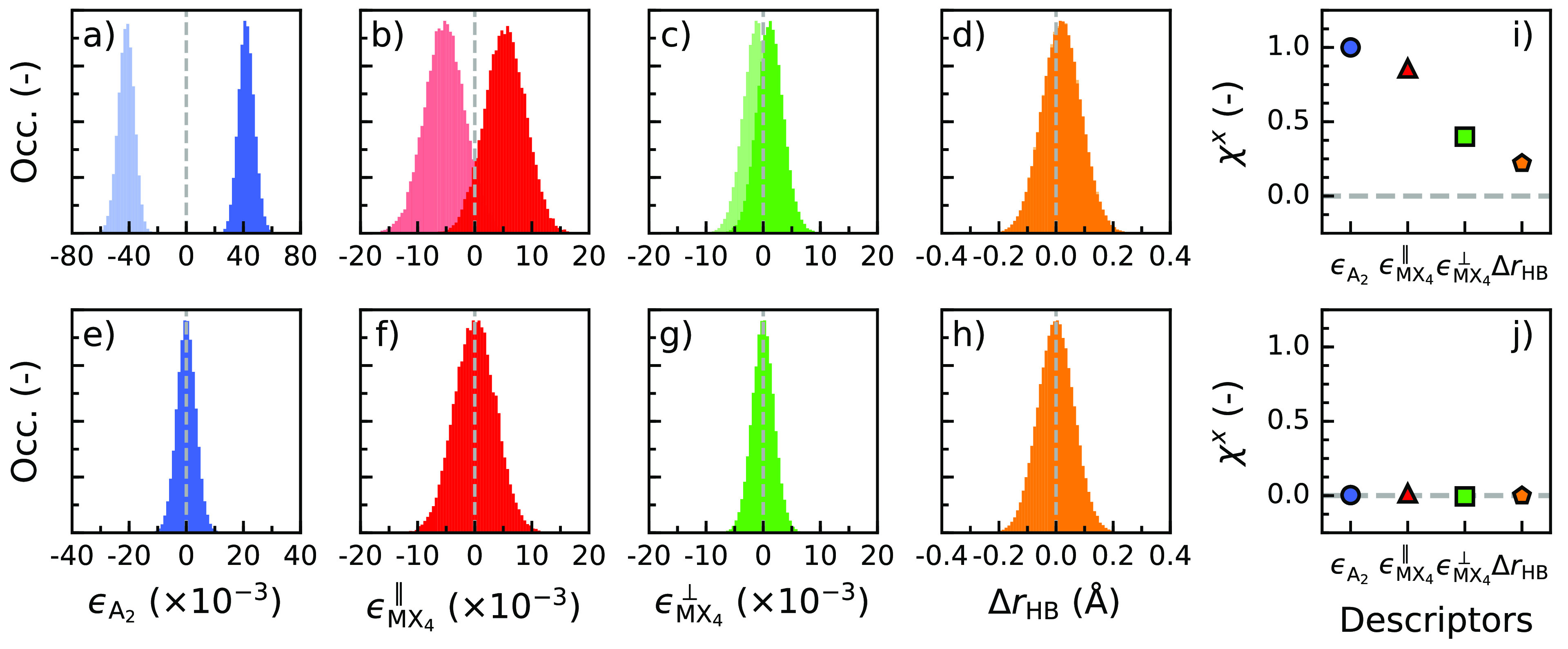
Finite-temperature distributions of structural descriptors and corresponding degree of chirality for MBA^+^-based perovskites at 50 K. Distributions of (a) cation chirality, (b) in-plane framework chirality, (c) out-of-plane framework chirality, and (d) hydrogen bond asymmetry of (*S*-MBA)_2_PbI_4_ and (*R*-MBA)_2_PbI_4_. Distributions of (e) cation chirality, (f) in-plane framework chirality, (g) out-of-plane framework chirality, and (h) hydrogen bond asymmetry of (*rac*-MBA)_2_PbI_4_. Degree of chirality for (i) (*S*-MBA)_2_PbI_4_ and (j) (*rac*-MBA)_2_PbI_4_. In panels a–d, the dark-colored distributions belong to (*S*-MBA)_2_PbI_4_, whereas the light-colored distributions correspond to (*R*-MBA)_2_PbI_4_.

To quantify the extent to which the chirality has decreased as a result of the temperature, we define a degree of chirality χ^*x*^ for a structural descriptor *x* as
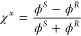
3with ϕ^*S*^ and ϕ^*R*^ representing the fractions of the distribution of *x* that we associate with the *S* and *R* enantiomers, respectively. Per definition, we have −1 ≤ χ^*x*^ ≤ +1, with a value of +1 (−1) corresponding to the ideal *S* form (*R* form) and a value of zero associated with an achiral structure. Intermediate values indicate a diminished chirality with a preference for one of the forms.

Figure 2i shows the degrees of chirality for the different structural descriptors of (*S*-MBA)_2_PbI_4_. Whereas the arrangement of cations (*ϵ*_A_2__) has the maximum degree of chirality χ^*x*^ = +1.00, the inorganic framework has considerably lower values with χ^*x*^ = +0.86 for the in-plane chirality (*ϵ*_MX_4__^∥^) and +0.40 for the out-of-plane chirality (*ϵ*_MX_4__^⊥^). As noted above, the hydrogen bond asymmetry (Δ*r*_HB_) suffers most from thermal motion, giving a small degree of chirality χ^*x*^ = +0.22. Again, as a check, in (*rac*-MBA)_2_PbI_4_, we find a negligible degree of chirality for all descriptors (χ^*x*^ ≈ 0), as shown in Figure 2j.

A clear trend emerges from the results shown in Figure 2. The chirality of the arrangement of organic cations is affected little by thermal motion at 50 K. In contrast, thermal vibrations in the inorganic framework limit the extent of the symmetry breaking and, thus, the degree of chirality in this framework. Concerning the latter, the axial halide anions, whose positions are used to determine *ϵ*_MX_4__^⊥^, are more mobile than the equatorial halide anions used to determine *ϵ*_MX_4__^∥^, explaining the smaller degree of chirality of the former compared to the latter. The hydrogen bonding between organic cations and the inorganic framework seems to be quite dynamic, resulting in a relatively small degree of chirality.

All of this indicates that the transfer of chirality from cations to an inorganic framework is incomplete and suffers from thermal motion. This can be studied in more detail by focusing on the temperature dependence of the chiral descriptors. To do so, we increase the temperature from 50 to 400 K in steps of 50 K and show the resulting distributions and corresponding degrees of chirality in Figure 3. While the qualitative trends established above in Figure 2 remain, we observe an increase in the broadening of the distributions with increasing temperatures because of increased thermal motion (panels a–d of Figure 3). For chiral perovskites, it implies that the degree of chirality χ^*x*^ decreases with an increasing temperature (Figure 3i). As a consistency check, we submitted achiral (*rac*-MBA)_2_PbI_4_ to the same procedure. It shows a similar temperature broadening of the distributions (panels e–h of Figure 3), but the degree of chirality for all descriptors remains zero (Figure 3j).

**Figure 3:**
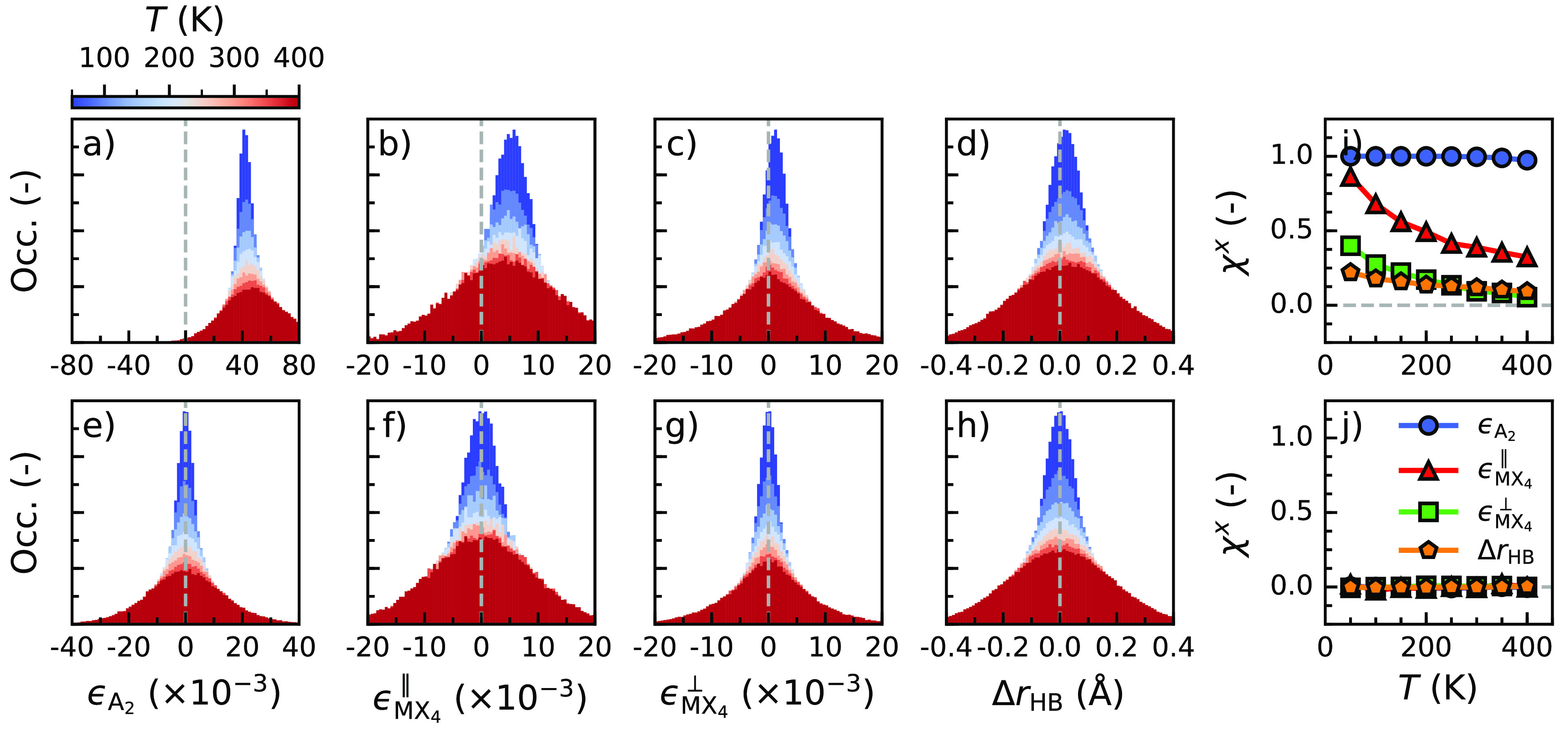
Temperature dependence of the degree of chirality in MBA^+^-based perovskites. Temperature-dependent descriptor distributions for (a) cation chirality, (b) in-plane framework chirality, (c) out-of-plane framework chirality, and (d) hydrogen bond asymmetry in (*S*-MBA)_2_PbI_4_. Temperature-dependent descriptor distributions for (e) cation chirality, (f) in-plane framework chirality, (g) out-of-plane framework chirality, and (h) hydrogen bond asymmetry in (*rac*-MBA)_2_PbI_4_. Temperature-dependent degree of chirality for (i) (*S*-MBA)_2_PbI_4_ and (j) (*rac*-MBA)_2_PbI_4_.

Interestingly, the degree of chirality decreases to a different extent for the various descriptors in (*S*-MBA)_2_PbI_4_. The chirality of the arrangement of organic cations (*ϵ*_A_2__) maintains a high degree of chirality for all investigated temperatures, only dropping to χ^*x*^ = +0.97 at 400 K. In contrast, the in-plane framework chirality (*ϵ*_MX_4__^∥^) drops to a significantly lower degree of chirality of χ^*x*^ = +0.32, whereas the out-of-plane framework chirality (*ϵ*_MX_4__^⊥^) almost vanishes at χ^*x*^ = +0.05 at 400 K. Likewise, the hydrogen bond asymmetry (Δ*r*_HB_), which already had a low degree of chirality at low temperatures, drops to a small value of χ^*x*^ = +0.09.

On the basis of this, we conclude that the arrangement of cations is rather insensitive to the temperature, whereas the in-plane chirality and out-of-plane chirality of the inorganic framework are considerably more sensitive, exhibiting a major drop with an increasing temperature. We attribute this to a decrease in chirality transfer from the organic cations to the inorganic framework with an increasing temperature. A more detailed analysis of the temperature dependence, in particular, its sensitivity to the type of structural enantiomer and the exchange–correlation functional that the MLFF is trained against, is given in Note 7 of the Supporting Information.

For a detailed understanding of the chirality transfer between the organic cations and the inorganic framework, we examine the interface between these two components. From our simulations, we find, not surprisingly, that the organic cations are rigidly bonded frameworks, with their atoms making relatively small vibrations around their equilibrium structure at a finite temperature. The analysis above shows that even their arrangement and overall orientation change little as a function of the temperature. The main degrees of freedom within the organic cations, active at a relatively low temperature, are rotations around single bonds, where in the packed crystal structure rotations of the NH_3_^+^ head groups are the most accessible.

Therefore, we focus on the hydrogen bonds between the NH_3_^+^ groups of the organic cations and the neighboring halide ions. Specifically, we assess the persistence of the N–H···X hydrogen bonds as a function of increasing temperature, focusing on the orientational autocorrelation function of the N–H bonds (Figure 4a). This autocorrelation function *A*_NH_3_^+^_ is defined as
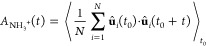
4with **û**_*i*_ = **r**_*i*_/|**r**_*i*_| being the normalized orientation vector of the *i*th N–H bond, *N* being the number of vectors used in the analysis, *t* being the time delay, and *t*_0_ being the time origin, where the autocorrelation function is averaged over different *t*_0_. Because the structures of the NH_3_^+^ groups are fairly rigid, a decay of *A*_NH_3_^+^_ is a clear sign of rotation of these groups. We define the orientation lifetime τ_NH_3_^+^_ as the time at which the autocorrelation function falls off to a value of *A*_NH_3_^+^_(τ_NH_3_^+^_) = *e*^–1^.

**Figure 4:**
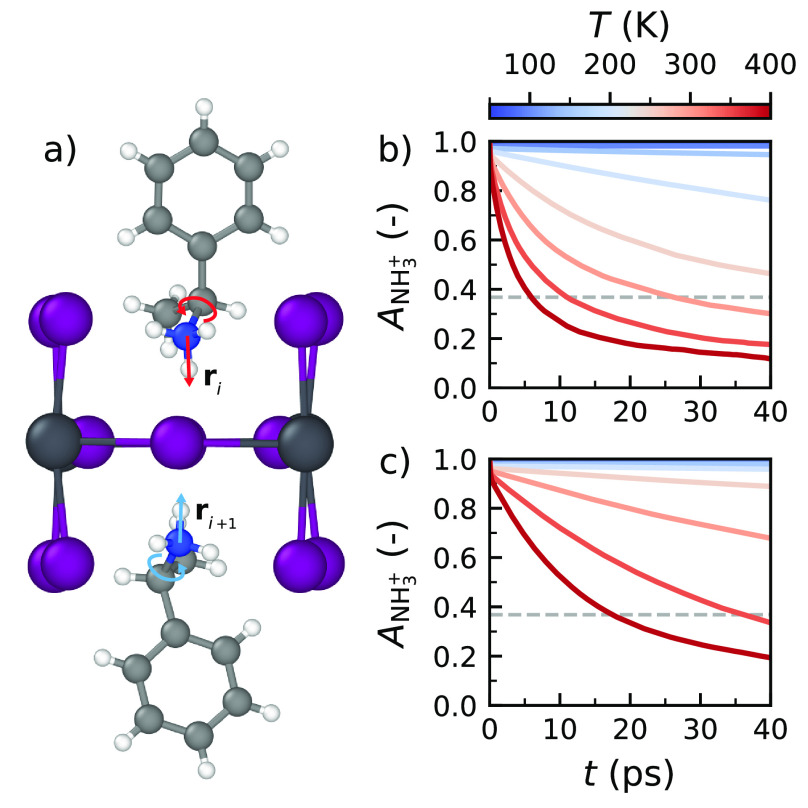
Orientational autocorrelation of the NH_3_^+^ headgroup of organic cations. (a) Schematic overview of the N–H bond vectors **r**_*i*_ used to determine the orientation of the headgroups of organic cations. Temporal autocorrelation of the headgroup orientation *A*_NH_3_^+^_ for the cations in (b) (*S*-MBA)_2_PbI_4_ and (c) (*rac*-MBA)_2_PbI_4_ at temperatures ranging from 50 to 400 K. The dashed gray line indicates where *A*_NH_3_^+^_ = *e*^–1^.

Evaluating the dynamics of the cation headgroups in chiral (*S*-MBA)_2_PbI_4_ (Figure 4b), we find that *A*_NH_3_^+^_ is constant at low temperatures (≤100 K), which indicates an absence of reorientations of the cation headgroups. For higher temperatures, we observe a decrease in *A*_NH_3_^+^_ and, thus, a reorientation of the N–H bonds in the perovskite. The typical time for such reorientations is τ_NH_3_^+^_ = 6.0 ps at 400 K, which indicates that the hydrogen bonds between the organic cations and the inorganic framework lose their structure at relatively short time scales. This lack of persistence in hydrogen bonding severely limits the ability of the cations to transfer their chirality to the inorganic framework, thus explaining the decreasing degree of chirality at elevated temperatures. We note that the autocorrelation functions do not decay to zero even after long times, which indicates a long time order in the orientation of the organic cations. This order is present as a result of the absence of any large-scale reorientations of the organic cations, as demonstrated using the width and length orientation vectors of the cations in Note 8 of the Supporting Information.

Similarly, one can monitor the autocorrelation functions in achiral (*rac*-MBA)_2_PbI_4_ (Figure 4c). A comparison to chiral (*S*-MBA)_2_PbI_4_ shows that the NH_3_^+^ headgroups in (*rac*-MBA)_2_PbI_4_ have a more persistent orientation, with headgroup reorientations only starting from 200 K and a typical reorientation time of τ_NH_3_^+^_ = 17.5 ps for the achiral structure at 400 K. We propose that the differences can be related to differences in the hydrogen bond strength in the two perovskites. The chiral perovskite structure is more distorted than the achiral structure and also has longer and, thus, weaker hydrogen bonds (Note 9 of the Supporting Information), as also discussed in refs (26 and 29).

In summary, we introduce a set of structural descriptors to characterize the chirality in 2D halide perovskites that are applicable to both static and finite temperature dynamical structures. The descriptors assess the chirality in the cations, the inorganic framework, and the asymmetry in the hydrogen bonds between the cations and inorganic layers. At finite temperatures, the structural descriptors no longer have a single value but instead exhibit a spread around average values as a result of thermal motion. If the distributions for descriptors referring to opposite enantiomeric crystals overlap, then the degree of chirality is reduced.

A comparison of the degree of chirality for the different structural descriptors reveals that the arrangement of the organic cations remains highly chiral up to a high temperature, whereas the chirality of the inorganic framework markedly decreases as a function of increasing temperature. Notably, a higher degree of chirality is observed for the in-plane direction than for the out-of-plane direction of the framework at elevated temperatures, which we explain through a larger mobility of the axial halide ions compared to that of the equatorial halide ions.

An analysis of the temperature dependence of the molecular motions reveals that, whereas the arrangement of organic cations is fairly stable with an increasing temperature, the NH_3_^+^ headgroups can rotate. The reorientations of these groups undermine the rigidity of the hydrogen bonding between the organic cations and the inorganic framework, which weakens the transfer of chirality between cations and the framework.

## Supporting Information Available

2D halide perovskite structures from experiments and DFT calculations (ZIP)
Additional structural descriptors for 2D halide perovskites, orientation vectors for cation chirality, computational settings for DFT calculations, additional details for structural descriptors, analysis of spin splitting in 2D halide perovskites, training procedure and validation of MLFFs, additional details for the degree of chirality, additional details on chirality transfer, and structural distortions and hydrogen bonds in perovskite structures (PDF)
