# Supplementary material for: Temperature-Dependent Chirality in Halide Perovskites
Source: J Phys Chem Lett. 2024 Jul 31;15(31):8057–64. doi: 10.1021/acs.jpclett.4c01629 (PMC11318036; doi:10.1021/acs.jpclett.4c01629)
Supplement: Supplementary file 2 — jz4c01629_si_002.pdf [file jz4c01629_si_002.pdf]

Supporting Information: Temperature-Dependent Chirality in Halide Perovskites

Mike Pols,[1,*] Geert Brocks,[1,2,†] Sofía Calero,[1,‡] and Shuxia Tao[1,§]

[1] *Materials Simulation & Modelling, Department of Applied Physics and Science Education, Eindhoven University of Technology, 5600 MB, Eindhoven, The Netherlands*

[2] *Computational Chemical Physics, Faculty of Science and Technology and MESA+ Institute for Nanotechnology, University of Twente, 7500 AE, Enschede, The Netherlands*

Keywords: metal halide perovskites, density functional theory, machine-learning force fields, chirality, chiral perovskites, structural descriptors, symmetry breaking, structural chirality, bond asymmetry, chirality transfer

* m.c.w.m.pols@tue.nl

† g.h.l.a.brocks@tue.nl

‡ s.calero@tue.nl

§ s.x.tao@tue.nl

CONTENTS

1. SI Note: Additional structural descriptor definitions S4
   A. Intraoctahedral distortions S4
   B. Interoctahedral distortions S5
   C. Planar distortions S6

2. SI Note: Cation orientation vectors S7
   A. Atomic fingerprints S7
   B. Orientation vectors S8

3. SI Note: Density functional theory S10

4. SI Note: Structural descriptors in 2D perovskites S15
   A. Chiral descriptors in 2D perovskites S15
   B. Components of in-plane framework chirality S17
   C. Effects of geometry optimization on structural descriptors S20
   D. Additional structural descriptors S21

5. SI Note: Spin-splitting in 2D perovskites S22

6. SI Note: Machine-learning force fields (MLFFs) S24
   A. Force field training S24
   B. Force field accuracy S26
   C. Molecular dynamics simulations S28

7. SI Note: Degree of chirality S30
   A. Uncertainty quantification of degree of chirality S30
   B. Temperature-dependence of chirality in $(R\text{-MBA})_2PbI_4$ S31
   C. Temperature-dependence of chirality in achiral perovskites S32
   D. Effects of exchange-correlation (XC) functional S33

8. SI Note: Chirality transfer S35
   A. Effects of exchange-correlation (XC) functional S35
   B. Cation reorientations S36

9. SI Note: Distortions and hydrogen bonds in perovskites S37

References S38

1. SI NOTE: ADDITIONAL STRUCTURAL DESCRIPTOR DEFINITIONS

In addition to the structural descriptors treated in the main text, we analyzed the two-dimensional (2D) perovskite structures with a range of additional descriptors that can be found in literature. The descriptors capture a variety of structural characteristics (Figure S1), including distortions of the inorganic octahedra (intraoctahedral), the asymmetry of the inorganic cages in the layers of the inorganic framework (interoctahedral), and distortions of the inorganic species away from the inorganic lattice planes (planar). In the following sections it is detailed how each of these structural descriptors are computed. Results regarding these descriptors applied to 2D perovskites are shown in SI Note 4 D.

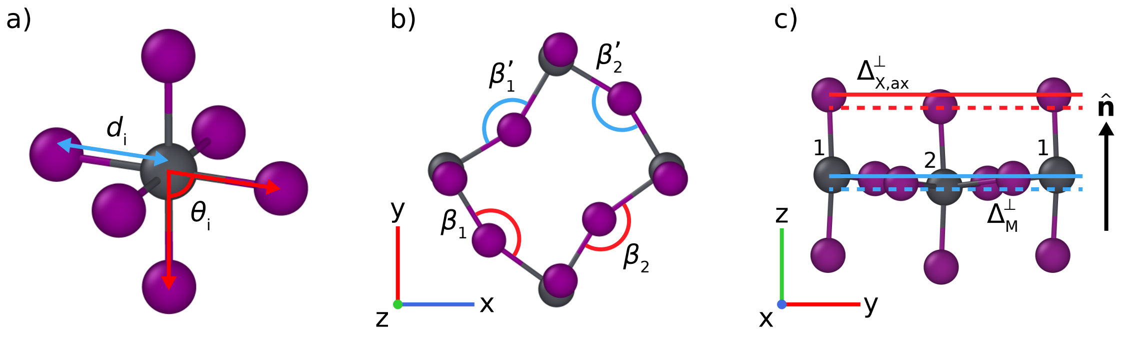

FIG. S1. Additional structural descriptors used to characterize 2D perovskite structures. (a) Intraoctahedral distortions describing the variance in M − X bond lengths and X − M − X bond angles. (b) Interoctahedral distortions that capture the cage asymmetry in the inorganic framework. (c) Planar distortions of the inorganic species away from the inorganic plane.

A. Intraoctahedral distortions

The distortions of the octahedra in the inorganic framework are quantified using metrics introduced by Robinson *et al.* [1] for the bond length and bond angle distortion. The bond length distortion Δd is calculated as

$$\Delta d = \frac{1}{6}\sum_{i=1}^{6}\left(\frac{d_i - d_0}{d_0}\right)^2 \quad (1)$$

with d_i the bond length of one of the six $\mathrm{M-X}$ bonds and d_0 the average length of all $\mathrm{M-X}$ bonds in the MX_6 octahedron. To determine the bond angle variance σ^2 we compute

$$\sigma^2 = \frac{1}{11}\sum_{i=1}^{12}\left(\theta_i - \theta_0\right)^2 , \tag{2}$$

with θ_i one of the twelve *cis* $\mathrm{X-M-X}$ bond angles and $\theta_0 = 90^\circ$ the ideal value for such bond angles. An overview of the bonds and angles used in this computation is shown in Figure S1a.

B. Interoctahedral distortions

To probe the asymmetry of the inorganic cages we employ the disparity in adjacent angles of the inorganic cage as introduced by Jana *et al.* [2, 3]. The bond angle disparity $\Delta\beta$ is computed as

$$\Delta\beta = \beta - \beta' \tag{3}$$

where β and β' ($\beta > \beta'$) are two unique $\mathrm{M-X-M}$ bond angles in a single inorganic cage. In static structures (Figure S1b), the presence of two unique angles $\beta = \beta_1 = \beta_2$ and $\beta' = \beta'_1 = \beta'_2$ is enough to break the inversion symmetry, however during finite temperature simulations the thermal fluctuations distort the internal $\mathrm{M-X-M}$ angles, resulting in four unique angles: β_1, β_2, β'_1 and β'_2. To counteract these thermal fluctuations, we take the average value of the angle pairs

$$\begin{aligned}\bar{\beta} &= \left(\beta_1 + \beta_2\right)/\,2\\ \bar{\beta}' &= \left(\beta'_1 + \beta'_2\right)/\,2\end{aligned} \tag{4}$$

and consequently compute an averaged bond angle disparity using the following relation

$$\Delta\bar{\beta} = \bar{\beta} - \bar{\beta}'. \tag{5}$$

Although the above-mentioned bond angle disparity was computed for $\mathrm{M-X-M}$ angles in general, it should be noted that in-plane ($\Delta\beta_{\mathrm{in}}$) and out-of-plane projections ($\Delta\beta_{\mathrm{out}}$) can also be computed using the formulas derived by Jana *et al.* [3].

Finally, using the values of the $\mathrm{M-X-M}$ angles in the inorganic cage, we can assign a value for the maximum distortion ($\max D$) of the inorganic cage. This is done by selecting the smallest angle from the set of all angles $\{\beta_1, \beta_2, \beta'_1, \beta'_2\}$ and checking its deviation from 180° as

$$\max D = 180^\circ - \min\left(\{\beta_1, \beta_2, \beta'_1, \beta'_2\}\right). \tag{6}$$

C. Planar distortions

To describe the breaking of symmetry in the inorganic layers, we employ planar distortion descriptors as introduced by Apergi *et al.* [4]. This symmetry breaking results from the existence of two unique octahedral sublattices in chiral halide perovskites (Figure S1c). The planar distortion $\Delta^{\perp}$ is computed by making use of these two unique sublattices as

$$\Delta^{\perp} = (\mathbf{r}_1 - \mathbf{r}_2) \cdot \hat{\mathbf{n}} = r_1^{\perp} - r_2^{\perp} \tag{7}$$

where $\mathbf{r}_1$ and $\mathbf{r}_2$ are the position vectors of a species in octahedral sublattice 1 and 2, $\hat{\mathbf{n}}$ is the unit normal vector to the inorganic layer, and $r_1^{\perp}$ and $r_2^{\perp}$ are the position vectors projected on the unit normal vector. The planar distortion can be calculated for a variety of species, in this work we compute it for the metal ions M ($\Delta^{\perp}_{\mathsf{M}}$) and axial halide species X_{ax} ($\Delta^{\perp}_{\mathsf{X}_{\mathsf{ax}}}$).

2. SI NOTE: CATION ORIENTATION VECTORS

A. Atomic fingerprints

To identify specific types of atoms in the topology of organic compounds (Figure S2a), we assign a unique string to every atom in organic compounds through a process we refer to as fingerprinting. The identification string or atomic fingerprint is based on the local environment of each atom. Given an appropriate descriptor for the topology of the organic compound, all non-equivalent atoms in a compound can be uniquely identified, thus allowing for a straightforward method to identify atoms and orientation vectors in the molecules in the crystal structure.

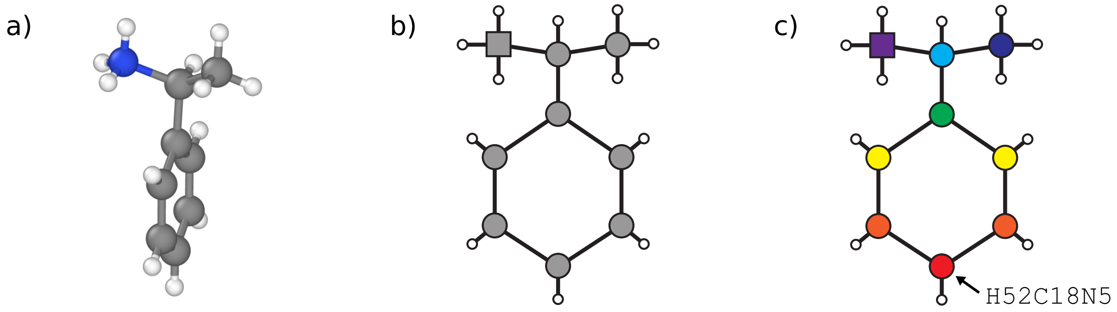

FIG. S2. Demonstration of atomic fingerprinting in MBA^+ cation. (a) Atomic structure of MBA^+ cation (H = white sphere; C = gray sphere, and N = blue sphere). (b) Graph representation of MBA^+ cation in which the nodes represent atoms and the edges represent bonds (H = white circle, C = gray circle, and N = gray square). (c) Graph representation of MBA^+ cation with color coding of the C and N atoms according to their atomic fingerprint; C and N atoms with the same color have identical atomic fingerprints.

Here, we assign atomic fingerprints using a graph representation of organic compounds (Figure S2b), which we construct using the connectivity matrix of the atoms. The nodes of the graph are represented by the atoms themselves and the edges represent the bonds between the atoms. Using the molecular graph, a bond-based distance matrix of the graph is constructed, describing how many bonds are needed to connect two species with each other. To prevent expensive computations for large compounds, the computation of the bond-based distance matrix can be truncated up to a cutoff N_{bonds} beyond which species are not considered connected. Using the

bond-based distance matrix, we compute the elemental distance sum for every atom ζ as

$$\zeta_i = \zeta_i^s = \left(\zeta_i^{s_1}, \ldots, \zeta_i^{s_{N_\mathrm{s}}}\right) = \sum_{j=1}^{N_\mathrm{bonds}} j \cdot N_{i,j}^s \tag{8}$$

with i iterating over the atoms in the system, s iterating over the different species in the compounds, j iterating over the various bond distances up to the cutoff N_bonds. $N_{i,j}^s$ represents the number of atoms of species s found at a bond distance j from the ith atom. The result of the procedure is that every atom is assigned a vector with N_s dimensions (Figure S2c). For ease-of-use these vectors were converted to a readable format by concatenating the elemental symbols with the elemental distance sums for all species as

$$\texttt{H52C18N5} \leftarrow \begin{cases} \zeta_i & = (52,\ 18,\ 5) \\ \mathrm{s} & = (\mathrm{H},\ \mathrm{C},\ \mathrm{N}) \end{cases} \tag{9}$$

as is shown in Figure S2c. Using this method with the MBA^+ cation, a total of 13 unique atomic fingerprints were assigned in the cation; 1 for N, 6 for C and 6 for H. The atomic fingerprints were subsequently used to extract a variety of internal vectors from the organic cations in the halide perovskites.

B. Orientation vectors

An evaluation of the chirality of the arrangement of cations requires the definition of cation orientation vectors. With a wide range of atoms to choose from, it is important that the chosen orientation vectors are uniquely defined through the atomic fingerprints of the organic cation. The orientation vectors used in this work are shown in Figure S3 for which the atom fingerprints shown in Table S1 were used to extract them from the perovskite crystal structures.

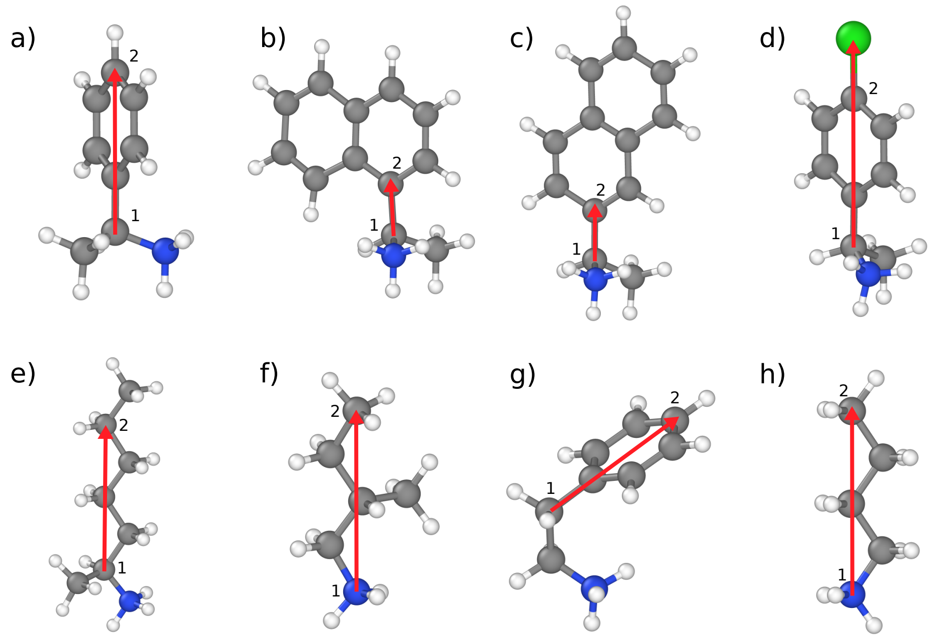

FIG. S3. Orientation vectors for organic cations in 2D halide perovskites used to compute the chirality of the cation arrangement. The orientation vectors for the (a) MBA^+, (b) $1NEA^+$, (c) $2NEA^+$, (d) 4-Cl-MBA^+, (e) 1-Me-HA^+, (f) 2-Me-BuA^+, (g) PEA^+, and (h) BA^+ cation are indicated with red arrows.

TABLE S1. Atom fingerprints used to determine the orientation vectors of various cations.

Cation	Element 1	Atom fingerprint 1	Element 2	Atom fingerprint 2
MBA^+	C	H32C16N1	C	H52C18N5
$1NEA^+$	C	H45C32N1	C	H45C24N2
$2NEA^+$	C	H47C36N1	C	H47C28N2
4-Cl-MBA^+	C	H27C16N1Cl5	Cl	H62C26N6Cl0
1-Me-HA^+	C	H59C16N1	C	H67C16N5
2-Me-BuA^+	N	H45C13N0	C	H45C9N4
PEA^+	C	H34C16N2	C	H54C18N6
BA^+	N	H36C10N0	C	H36C6N4

3. SI NOTE: DENSITY FUNCTIONAL THEORY

To investigate the structural characteristics of the 2D perovskites, we carried out density functional theory (DFT) calculations for a variety of experimental crystal structures (Table S2). We performed these DFT calculations with the Vienna Ab-initio Simulation Package (VASP) [5–7] and modelled the electron-electron exchange interaction either using the SCAN [8] or PBE+D3(BJ) [9, 10] functionals. The projector-augmented wave (PAW) pseudopotentials [11] treated the following electrons: H ($1s^1$), C ($2s^2 2p^2$), N ($2s^2 2p^3$), Cl ($3s^2 3p^5$), Br ($4s^2 4p^5$), I ($5s^2 5p^5$), and Pb ($6s^2 6p^2$) as valence electrons. The cutoff energy for the plane wave basis set was set to 500 eV in all calculations, combined with convergence criteria for the forces and energies of 1×10^{-5} eV and 1×10^{-2} eV Å^{-1}, respectively. The reciprocal space was sampled using a Γ-centered k-mesh [12]. In optimizing the experimental crystal structures all atomic positions, the cell shape and cell volume were allowed to change. The cell parameters of the experimental structures and the structures optimized using DFT calculations are shown in Table S3, Table S4, and Table S5. We highlight that the SCAN functional performs better for the structures of the 2D perovskites than the PBE+D3(BJ) functional. SCAN predicts a unit cell volume of on average -1.5% below that from experiments, whereas PBE+D3(BJ) underpredicts the unit cell volume with -3.2%.

TABLE S2. Experimental crystal structures of investigated 2D halide perovskites with their space group and space group number, characterization temperature and database identification number.

Perovskite	Space group (N_{group})	$T_{\mathrm{exp.}}$ (K)	Database ID	References
(*S*-MBA)$_2$PbI$_4$	$P2_12_12_1$ (19)	298	CCDC:2015617	Ref.[2]
(*rac*-MBA)$_2$PbI$_4$	$P2_1/c$ (14)	293	CCDC:1877052	Ref.[13]
(*R*-1NEA)$_2$PbBr$_4$	$P2_1$ (4)	298	CCDC:2015620	Ref.[2]
(*S*-1NEA)$_2$PbBr$_4$	$P2_1$ (4)	298	CCDC:2015618	Ref.[2]
(*rac*-1NEA)$_2$PbBr$_4$	$P2_1/c$ (14)	298	CCDC:2015614	Ref.[2]
(*R*-2NEA)$_2$PbBr$_4$	$P2_1$ (4)	298	CCDC:2178604	Ref.[14]
(*S*-2NEA)$_2$PbBr$_4$	$P2_1$ (4)	298	CCDC:2178605	Ref.[14]
(*rac*-2NEA)$_2$PbBr$_4$	$P2_1/c$ (14)	298	CCDC:2178606	Ref.[14]
(*R*-4-Cl-MBA)$_2$PbBr$_4$	$P2_12_12_1$ (19)	299.4	CCDC:2095482	Ref.[3]
(*S*-1-Me-HA)$_2$PbI$_4$	$P2_12_12_1$ (19)	298.76	CCDC:2095483	Ref.[3]
(*S*-2-Me-BuA)$_2$PbBr$_4$	$P2_1$ (4)	297.5	CCDC:2095485	Ref.[3]
PEA$_2$PbI$_4$	$P\bar{1}$ (2)	296	CCDC:1542461	Ref.[15]
BA$_2$PbI$_4$	$Pbca$ (61)	100	CCDC:2018893	Ref.[16]

TABLE S3. Experimental and DFT-optimized crystal geometries for 2D halide perovskites.

Perovskite	Type	a (Å)	b (Å)	c (Å)	α (°)	β (°)	γ (°)	V (Å^3)	k-points
	Exp.	8.90	28.86	9.31	90.0	90.0	90.0	2393.31	
(S-MBA)$_2$PbI$_4$	SCAN	8.87	28.76	9.19	90.0	90.0	90.0	2342.49	2×1×2
	PBE+D3(BJ)	8.76	28.53	9.12	90.0	90.0	90.0	2279.16	
	Exp.	14.62	9.38	8.78	90.0	100.1	90.0	1185.67	
(*rac*-MBA)$_2$PbI$_4$	SCAN	14.66	9.35	8.66	90.0	100.8	90.0	1166.97	2×2×2
	PBE+D3(BJ)	14.56	9.28	8.55	90.0	99.9	90.0	1136.78	
	Exp.	8.74	8.74	33.00	84.6	84.7	89.6	2498.29	
PEA$_2$PbI$_4$	SCAN	8.70	8.72	32.76	85.6	85.7	89.4	2471.51	2×2×1
	PBE+D3(BJ)	8.61	8.61	32.36	85.8	85.8	89.3	2386.81	
	Exp.	8.42	9.00	26.08	90.0	90.0	90.0	1975.59	
BA$_2$PbI$_4$	SCAN	8.45	9.00	26.10	90.0	90.0	90.0	1986.58	2×2×1
	PBE+D3(BJ)	8.34	8.96	26.00	90.0	90.0	90.0	1943.34	

TABLE S4. Experimental and DFT-optimized crystal geometries for 2D halide perovskites.

Perovskite	Type	a (Å)	b (Å)	c (Å)	α (°)	β (°)	γ (°)	V (Å^3)	k-points
	Exp.	8.76	7.96	19.52	90.0	93.8	90.0	1357.79	
(R-1NEA)$_2$PbBr$_4$	SCAN	8.67	7.91	19.40	90.0	94.4	90.0	1325.75	2×2×1
	PBE+D3(BJ)	8.63	7.79	19.38	90.0	93.9	90.0	1299.72	
	Exp.	8.75	7.96	19.50	90.0	93.8	90.0	1355.17	
(S-1NEA)$_2$PbBr$_4$	SCAN	8.67	7.91	19.42	90.0	94.5	90.0	1326.54	2×2×1
	PBE+D3(BJ)	8.62	7.82	19.38	90.0	94.0	90.0	1303.01	
	Exp.	19.25	8.08	8.73	90.0	90.3	90.0	1357.21	
(rac-1NEA)$_2$PbBr$_4$	SCAN	19.10	8.07	8.68	90.0	90.9	90.0	1337.41	1×2×2
	PBE+D3(BJ)	19.14	7.96	8.63	90.0	90.9	90.0	1314.26	
	Exp.	8.77	7.84	20.35	90.0	99.5	90.0	1380.98	
(R-2NEA)$_2$PbBr$_4$	SCAN	8.70	7.78	20.33	90.0	99.7	90.0	1356.31	2×2×1
	PBE+D3(BJ)	8.68	7.67	20.29	90.0	100.1	90.0	1329.32	
	Exp.	8.78	7.84	20.34	90.0	99.5	90.0	1380.50	
(S-2NEA)$_2$PbBr$_4$	SCAN	8.69	7.79	20.30	90.0	99.7	90.0	1354.53	2×2×1
	PBE+D3(BJ)	8.68	7.67	20.27	90.0	100.1	90.0	1327.93	
	Exp.	20.13	7.91	8.74	90.0	92.5	90.0	1390.86	
(rac-2NEA)$_2$PbBr$_4$	SCAN	19.98	7.80	8.69	90.0	92.3	90.0	1352.83	1×2×2
	PBE+D3(BJ)	19.92	7.68	8.68	90.0	91.1	90.0	1329.32	

TABLE S5. Experimental and DFT-optimized crystal geometries for 2D halide perovskites.

Perovskite	Type	a (Å)	b (Å)	c (Å)	α (°)	β (°)	γ (°)	V (Å^3)	k-mesh
	Experiment	7.91	8.81	35.58	90.0	90.0	90.0	2479.45	
(R-4-Cl-MBA)$_2$PbBr$_4$	SCAN	7.86	8.71	35.60	90.0	90.0	90.0	2438.11	2×2×1
	PBE+D3(BJ)	7.80	8.69	35.29	90.0	90.0	90.0	2390.75	
	Experiment	8.97	8.97	33.78	90.0	90.0	90.0	2717.76	
(S-1-Me-HA)$_2$PbI$_4$	SCAN	8.88	8.88	33.93	90.0	90.0	90.0	2674.37	2×2×1
	PBE+D3(BJ)	8.80	8.78	33.82	90.0	90.0	90.0	2615.19	
	Experiment	15.69	8.29	8.24	90.0	101.2	90.0	1050.28	
(S-2-Me-BuA)$_2$PbBr$_4$	SCAN	15.29	8.21	8.18	90.0	101.4	90.0	1006.72	2×2×2
	PBE+D3(BJ)	15.36	8.13	8.13	90.0	101.0	90.0	996.44	

4. SI NOTE: STRUCTURAL DESCRIPTORS IN 2D PEROVSKITES

A. Chiral descriptors in 2D perovskites

In addition to the chiral 2D perovskites shown in the main text, we also used the structural descriptors to evaluate the chirality in a variety of other perovskites. Here, we analyze the structural chirality of a variety of 2D perovskites found in previous works in literature shown in Table S2 [2, 3, 13–16], which we optimized using DFT calculations. The results from this analysis can be found in Table S6, Table S7, and Table S8. The overview highlights the descriptors can distinguish between chiral and achiral perovskites as well as the handedness of the various components in the crystal structures of the 2D perovskites, as evidenced by the values and signs of the structural descriptors. We note that we mirrored the (S-MBA)$_2$PbI$_4$ structure to obtain the (R-MBA)$_2$PbI$_4$ structure, resulting in exact mirror images. As a result of this, the values of the structural chirality descriptors are of equal magnitude but opposite sign. In contrast, independent crystal structures for both enantiomers were provided in the literature for NEA$^+$-based perovskites. These structures are not exactly identical mirror images, resulting in slight differences in the absolute values of the descriptors.

TABLE S6. Structural descriptors for chirality and bond asymmetry in 2D perovskite structures.

Perovskite	ϵ_{A_2} $(\times 10^{-3})$	$\epsilon^{\parallel}_{\mathsf{MX}_4}$ $(\times 10^{-3})$	$\epsilon^{\perp}_{\mathsf{MX}_4}$ $(\times 10^{-3})$	Δr_{HB} (Å)
(S-MBA)$_2$PbI$_4$	+46.490	+4.534	+1.560	0.036
(R-MBA)$_2$PbI$_4$	-46.490	-4.534	-1.560	0.036
(rac-MBA)$_2$PbI$_4$	0.000	0.000	0.000	0.000
PEA$_2$PbI$_4$	0.000	0.000	0.000	0.000
BA$_2$PbI$_4$	0.000	0.000	0.000	0.000

TABLE S7. Structural descriptors for chirality and bond asymmetry in 2D perovskite structures.

Perovskite	ϵ_{A_2} ($\times 10^{-3}$)	$\epsilon^{\parallel}_{MX_4}$ ($\times 10^{-3}$)	$\epsilon^{\perp}_{MX_4}$ ($\times 10^{-3}$)	Δr_{HB} (Å)
(*S*-1NEA)$_2$PbBr$_4$	-4.341	-13.105	+9.172	0.044
(*R*-1NEA)$_2$PbBr$_4$	+4.481	+12.629	-9.319	0.058
(*rac*-1NEA)$_2$PbBr$_4$	0.000	0.000	0.000	0.000
(*S*-2NEA)$_2$PbBr$_4$	+10.701	+5.519	+1.787	0.098
(*R*-2NEA)$_2$PbBr$_4$	-10.791	-4.394	-2.021	0.107
(*rac*-2NEA)$_2$PbBr$_4$	0.000	0.000	0.000	0.000

TABLE S8. Structural descriptors for chirality and bond asymmetry in 2D perovskite structures.

Perovskite	ϵ_{A_2} ($\times 10^{-3}$)	$\epsilon^{\parallel}_{MX_4}$ ($\times 10^{-3}$)	$\epsilon^{\perp}_{MX_4}$ ($\times 10^{-3}$)	Δr_{HB} (Å)
(*R*-4-Cl-MBA)$_2$PbBr$_4$	-21.903	+7.762	+0.229	0.031
(*S*-1-Me-HA)$_2$PbI$_4$	-13.333	+13.230	-0.648	0.033
(*S*-2-Me-BuA)$_2$PbBr$_4$	+38.809	-4.302	+0.849	0.000

B. Components of in-plane framework chirality

As indicated in the manuscript, the in-plane framework chirality ($\epsilon^{\parallel}_{\mathsf{MX_4}}$) can be decomposed into various components. By determining the chirality in the direction of the axes spanning the inorganic layers, we obtain chirality components that probe the structural chirality along those directions (Table S9). An example of such a decomposition of the in-plane framework chirality is shown for (*S*-1NEA)$_2$PbBr$_4$ in Figure S4. The net chirality in the axial directions ($\epsilon^{\mathrm{ax},1}_{\mathsf{MX_4}}$ and $\epsilon^{\mathrm{ax},2}_{\mathsf{MX_4}}$) is obtained by averaging the various unique chirality values in those directions. For (*S*-1NEA)$_2$PbBr$_4$ we find two unique values for the framework chirality in the direction parallel to the 2_1 screw axis (b-axis). The two values have an opposite sign and magnitude ($\epsilon^{\mathrm{ax},1(1)}_{\mathsf{MX_4}}$ = -143.524×10^{-3} and $\epsilon^{\mathrm{ax},1(2)}_{\mathsf{MX_4}}$ = +71.620×10^{-3}), thus resulting in a net nonzero chirality in this direction of $\epsilon^{\mathrm{ax},1}_{\mathsf{MX_4}}$ = -35.952×10^{-3} in the direction of the b-axis. Along the other axis, the a-axis, we find only a single value for the chirality ($\epsilon^{\mathrm{ax},2(1)}_{\mathsf{MX_4}} = \epsilon^{\mathrm{ax},2(2)}_{\mathsf{MX_4}}$ = +14.691×10^{-3}) which is thus also the net chirality of the inorganic layers in this direction, $\epsilon^{\mathrm{ax},2}_{\mathsf{MX_4}}$ = +14.691×10^{-3}. We find the sum of the net chirality in the two axial directions roughly approximates the in-plane framework chirality for all investigated perovskites ($\epsilon^{\mathrm{ax},1}_{\mathsf{MX_4}} + \epsilon^{\mathrm{ax},2}_{\mathsf{MX_4}} \approx \epsilon^{\parallel}_{\mathsf{MX_4}}$), thus indicating the in-plane framework chirality appropriately probes the chirality in all directions of the inorganic layers. Finally, we note that in achiral perovskites, such as (*rac*-1NEA)$_2$PbBr$_4$ and (*rac*-2NEA)$_2$PbBr$_4$, one does find nonzero chirality values in the inorganic layers. However, due to an opposite sign and identical magnitude, there is a nonzero net chirality in the axial directions in the inorganic layers.

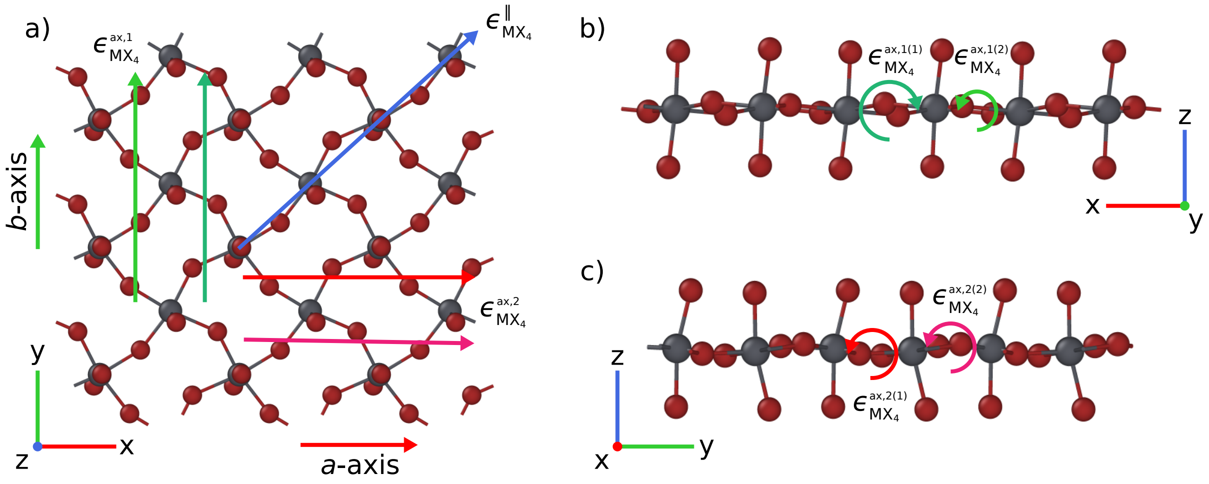

FIG. S4. Decomposition of in-plane framework chirality into components along axial directions. (a) Overview of the various directions and framework chirality considered in $(S\text{-1NEA})_2PbBr_4$. (b) Two unique chirality values are found in the direction of the b-axis that is parallel to the 2_1 screw axis and (c) only a single value in the direction of the a-axis.

TABLE S9. Components of the in-plane framework chirality in 2D perovskite structures.

Perovskite	$\epsilon^{\parallel}_{MX_4}$ ($\times 10^{-3}$)	$\epsilon^{ax,1}_{MX_4}$ ($\times 10^{-3}$)	Direction 1* $\epsilon^{ax,1(1)}_{MX_4}$ ($\times 10^{-3}$)	$\epsilon^{ax,1(2)}_{MX_4}$ ($\times 10^{-3}$)	$\epsilon^{ax,2}_{MX_4}$ ($\times 10^{-3}$)	Direction 2* $\epsilon^{ax,2(1)}_{MX_4}$ ($\times 10^{-3}$)	$\epsilon^{ax,2(2)}_{MX_4}$ ($\times 10^{-3}$)
(*S*-1NEA)$_2$PbBr$_4$	-13.105	-35.952	-143.524	+71.620	+14.691	+14.691	+14.691
(*R*-1NEA)$_2$PbBr$_4$	+12.629	+40.313	+150.501	-69.876	-19.375	-19.375	-19.375
(*rac*-1NEA)$_2$PbBr$_4$	0.000	0.000	0.000	0.000	0.000	+12.727	−12.727
(*S*-2NEA)$_2$PbBr$_4$	+5.519	+30.709	+143.512	-82.094	-21.166	-21.166	-21.166
(*R*-2NEA)$_2$PbBr$_4$	-4.394	-34.560	-147.671	+78.550	+26.102	+26.102	+26.102
(*rac*-2NEA)$_2$PbBr$_4$	0.000	0.000	0.000	0.000	0.000	+30.614	−30.614

C. Effects of geometry optimization on structural descriptors

In addition to the computation of the structural descriptors for structures optimized with the SCAN functional, we also determined the values of the structural descriptors for the experimental structure and the PBE+D3(BJ)-optimized structures of MBA_2PbI_4. The results of this analysis are shown in Table S10. The nature of the crystal structures appears to have only a small effect on the values of the structural descriptors, indicating that the descriptors are rather robust in identifying structural chirality in 2D perovskites.

TABLE S10. Effect of structural optimizations on structural chirality in I-based 2D perovskites.

Perovskite	Type	ϵ_{A_2} ($\times 10^{-3}$)	$\epsilon^{\parallel}_{MX_4}$ ($\times 10^{-3}$)	$\epsilon^{\perp}_{MX_4}$ ($\times 10^{-3}$)	Δr_{HB} (Å)
(S-MBA)$_2$PbI$_4$	Exp.	+49.313	+5.252	+1.341	0.029
	SCAN	+46.490	+4.534	+1.560	0.036
	PBE+D3(BJ)	+44.446	+5.917	+1.578	0.031
(R-MBA)$_2$PbI$_4$	Exp.	-49.313	-5.252	-1.341	0.029
	SCAN	-46.490	-4.534	-1.560	0.036
	PBE+D3(BJ)	-44.446	-5.917	-1.578	0.031
(rac-MBA)$_2$PbI$_4$	Exp.	0.000	0.000	0.000	0.000
	SCAN	0.000	0.000	0.000	0.000
	PBE+D3(BJ)	0.000	0.000	0.000	0.000

D. Additional structural descriptors

TABLE S11. Additional structural descriptors as determined for crystal geometries optimized with the SCAN functional.

Perovskite	σ^2 ($^{\circ 2}$)	Δd ($\times 10^{-5}$)	$\Delta\beta$ ($^\circ$)	$\Delta\beta_{\mathrm{in}}$ ($^\circ$)	$\Delta\beta_{\mathrm{out}}$ ($^\circ$)	$\max D$ ($^\circ$)	$\max D_{\mathrm{in}}$ ($^\circ$)	$\max D_{\mathrm{out}}$ ($^\circ$)	$\Delta^{\perp}_{\mathrm{M}}$ (Å)	$\Delta^{\perp}_{\mathrm{X_{ax}}}$ (Å)
(*S*-MBA)$_2$PbI$_4$	20.17	29.38	7.383	7.376	0.652	31.352	31.345	0.748	0.061	0.043
(*R*-MBA)$_2$PbI$_4$	20.17	29.38	7.383	7.376	0.652	31.352	31.345	0.748	0.061	0.043
(*rac*-MBA)$_2$PbI$_4$	14.04	18.19	0.000	0.000	0.000	26.193	26.191	0.293	0.000	0.000
PEA$_2$PbI$_4$	4.10	4.47	1.622	1.649	-1.180	31.476	31.476	1.623	0.000	0.009
BA$_2$PbI$_4$	7.29	0.54	0.000	0.000	0.000	32.933	23.964	23.250	0.000	0.000
(*S*-1NEA)$_2$PbBr$_4$	42.48	75.48	16.075	18.103	-3.781	39.957	39.402	11.113	0.142	0.126
(*R*-1NEA)$_2$PbBr$_4$	43.21	70.85	15.980	18.324	-4.554	39.968	39.443	11.688	0.135	0.111
(*rac*-1NEA)$_2$PbBr$_4$	22.58	20.88	0.000	0.000	0.000	27.844	27.826	1.056	0.000	0.000
(*S*-2NEA)$_2$PbBr$_4$	48.15	47.24	15.997	15.341	5.229	40.092	38.506	12.038	0.072	0.182
(*R*-2NEA)$_2$PbBr$_4$	49.53	48.14	15.946	15.078	6.024	40.555	38.863	12.517	0.058	0.170
(*rac*-2NEA)$_2$PbBr$_4$	23.43	8.97	0.000	0.000	0.000	31.970	31.878	2.550	0.000	0.000
(*R*-4-Cl-MBA)$_2$PbBr$_4$	52.46	72.28	14.266	10.782	14.690	39.383	35.729	17.771	0.113	0.201
(*S*-1-Me-HA)$_2$PbI$_4$	10.91	3.33	1.033	1.026	0.163	29.372	29.348	1.464	0.172	0.169
(*S*-2-Me-BuA)$_2$PbBr$_4$	10.55	2.56	1.861	1.827	0.955	30.025	29.984	1.663	0.052	0.078

5. SI NOTE: SPIN-SPLITTING IN 2D PEROVSKITES

To correlate the spin-splitting in 2D perovskites with the aforementioned structural descriptors, we analyze the spin-splitting in the band structure of the perovskites. In this analysis we employ the perovskite geometries optimized using the SCAN XC functional. Analogous to earlier work [3], we evaluate the spin-splitting of the lowest conduction bands in the Γ-X, Γ-Y and Γ-Z directions using the PBE XC functional including effects of spin-orbit coupling (SOC). Charge densities were converged with an energy convergence criterion of 1×10^{-8} eV on the same k-mesh used for the geometry optimizations.

We analyze the spin-splitting of the bands near the Γ-point through the following functional form [3]

$$E_{\pm}(\mathbf{k}) = \frac{\hbar^2 k^2}{2m} \pm \alpha_{\text{eff}} k \tag{10}$$

where E_+ and E_- are the energies of the spin-split bands and α_{eff} is the spin-splitting coefficient. This spin-splitting coefficient can be rewritten as

$$\alpha_{\text{eff}} = \frac{\Delta E_{\pm}}{2k_0} \tag{11}$$

with k_0 as the characteristic momentum offset of the spin-split bands and $\Delta E_{\pm}$ the energy splitting between the bands at k_0 (i.e. $\Delta E_{\pm} = E_+ - E_-$). The resulting spin-splitting parameters for the band exhibiting maximum spin-splitting are shown in Table S12.

TABLE S12. Spin-splitting parameters of chiral 2D perovskite structures.

Perovskite	k_0 (Å^{-1})	$\Delta E_{\pm}$ (eV)	α_{eff} (eV Å)
(S-MBA)$_2$PbI$_4$	0.026	0.044	0.841
(R-1NEA)$_2$PbBr$_4$	0.057	0.159	1.386
(R-2NEA)$_2$PbBr$_4$	0.051	0.124	1.201
(R-4-Cl-MBA)$_2$PbBr$_4$	0.032	0.041	0.647
(S-1-Me-HA)$_2$PbI$_4$	0.002	0.000	0.076
(S-2-Me-BuA)$_2$PbBr$_4$	0.006	0.002	0.155

In Table S13 we compare the effective spin-splitting parameter α_{eff} with a range of structural descriptors. The comparison indicates that the structural chirality of the inorganic framework ($\epsilon^{\parallel}_{\text{MX}_4}$ and $\epsilon^{\perp}_{\text{MX}_4}$) shows no clear correlation with the spin-splitting parameter. The hydrogen bond

asymmetry (Δr_{HB}) and in-plane bond angle disparity ($\Delta\beta_{\mathrm{in}}$) show somewhat more correlation with the spin-splitting.

TABLE S13. Effective spin-splitting parameter against a variety of structural descriptors of chiral 2D perovskite structures.

Perovskite	α_{eff} (eV Å)	$\epsilon^{\parallel}_{\mathrm{MX_4}}$ ($\times 10^{-3}$)	$\epsilon^{\perp}_{\mathrm{MX_4}}$ ($\times 10^{-3}$)	Δr_{HB} (Å)	$\Delta\beta_{\mathrm{in}}$ (°)
(S-MBA)$_2$PbI$_4$	0.841	+4.534	+1.560	0.036	7.376
(R-1NEA)$_2$PbBr$_4$	1.386	+12.629	-9.319	0.058	18.324
(R-2NEA)$_2$PbBr$_4$	1.201	-4.394	-2.021	0.107	15.078
(R-4-Cl-MBA)$_2$PbBr$_4$	0.647	+7.762	+0.229	0.031	10.782
(S-1-Me-HA)$_2$PbI$_4$	0.076	+13.230	-0.648	0.033	1.026
(S-2-Me-BuA)$_2$PbBr$_4$	0.155	-4.302	+0.849	0.000	1.827

6. SI NOTE: MACHINE-LEARNING FORCE FIELDS (MLFFS)

A. Force field training

For the investigation of the finite-temperature effects we trained machine-learning force fields (MLFFs) against total total energies, forces and stresses from DFT calculations. The training set was automatically constructed using an on-the-fly active learning scheme from dynamical simulations in an *NpT* ensemble [17]. Local atomic environments were described using an adaptation of the smooth overlap of atomic positions (SOAP) descriptor [18], for which we employed a cutoff for the two-body radial descriptor $\rho_i^{(2)}$ of 6.0 Å, and a 4.0 Å cutoff for the three-body angular descriptor $\rho_i^{(3)}$. The atomic positions were broadened using Gaussian distributions with a width of 0.5 Å. Both descriptors were expanded on a basis set of spherical Bessel functions and Legendre polynomials, using 8 and 6 Bessel functions for the two-body and three-body descriptors, respectively, with a maximum angular momentum quantum number of $l_{\mathrm{max}} = 2$. The expansion coefficients of this basis set constitute the descriptor for the local atomic environments. To measure the similarity between two local atomic environments, we employed a polynomial kernel function to a power 4, in which the two-body radial and three-body angular descriptor vectors were weighted by 0.1 and 0.9, respectively.

The training of the MLFFs started with a constant temperature simulation at 300 K, using the optimized crystal geometry as the starting point. The initial training run was followed by consecutive constant temperature simulations at 100 K and 450 K, each of which used the final positions and velocities of the previous run as starting point. For all constant temperature simulations the time of the training run was 50 ps. In the final training run we cooled the model systems from 350 K to 50 K over 60 ps, the starting point (i.e. positions and velocities) for these runs was obtained using 10 ps equilibration runs with the respective intermediate force fields. During training the temperature and pressure were controlled using Parinello-Rahman dynamics [19, 20] using friction coefficients $\gamma = 5\,\mathrm{ps}^{-1}$ and $\gamma_{\mathrm{L}} = 5\,\mathrm{ps}^{-1}$ for the atomic and lattice degrees of freedom, respectively. A time step of $\Delta t = 2\,\mathrm{fs}$ was used together with an increased mass of the hydrogen atoms of $m_{\mathrm{H}} = 4\,\mathrm{u}$, to enhance the sampling of structures. To limit the size of the force fields, the number of local reference configuration was limited to 2000, which was enforced by allowing configurations to be discarded once saturated. Using the training data of the final MLFFs, we refit the models onto faster descriptors without any Bayesian error

estimation to speed up the evaluation for large-scale molecular dynamics production runs. The number of structures in the training sets (N_{DFT}) and the number of local reference configuration for the different elements in the MLFFs (N_{basis}) can be found in Table S14.

TABLE S14. Size of the training set and number of local reference configurations for various machine-learning force fields (MLFFs) for 2D perovskites.

XC functional	Training structure	N_{DFT} (-)	N_{basis} (-)				
			H	C	N	I	Pb
SCAN	$(S\text{-MBA})_2PbI_4$	972	2000	2000	563	1240	310
	PEA_2PbI_4	1030	2000	2000	507	1175	218
	BA_2PbI_4	973	2000	1983	471	1129	226
PBE+D3(BJ)	$(S\text{-MBA})_2PbI_4$	933	2000	2000	568	1190	283

B. Force field accuracy

To check the ability of the MLFFs to reproduce the forces from DFT calculations, we run short simulations in which we heated the systems used during training from 50 K to 450 K during 80 ps using the final MLFFs obtained from training. For these short simulations we decreased the timestep to $\Delta t = 1\,\mathrm{fs}$ whilst decreasing the hydrogen mass to $m_\mathrm{H} = 1\,\mathrm{u}$. The resulting trajectories were divided into 40 equal parts each spanning a temperature range of 10 K. From each of these parts we randomly selected one frame and evaluated it using both the MLFF and DFT calculations, for which we compared the force components of all atoms in Figure S5. All models show a high correlation and low error between the MLFF forces and those obtained using DFT.

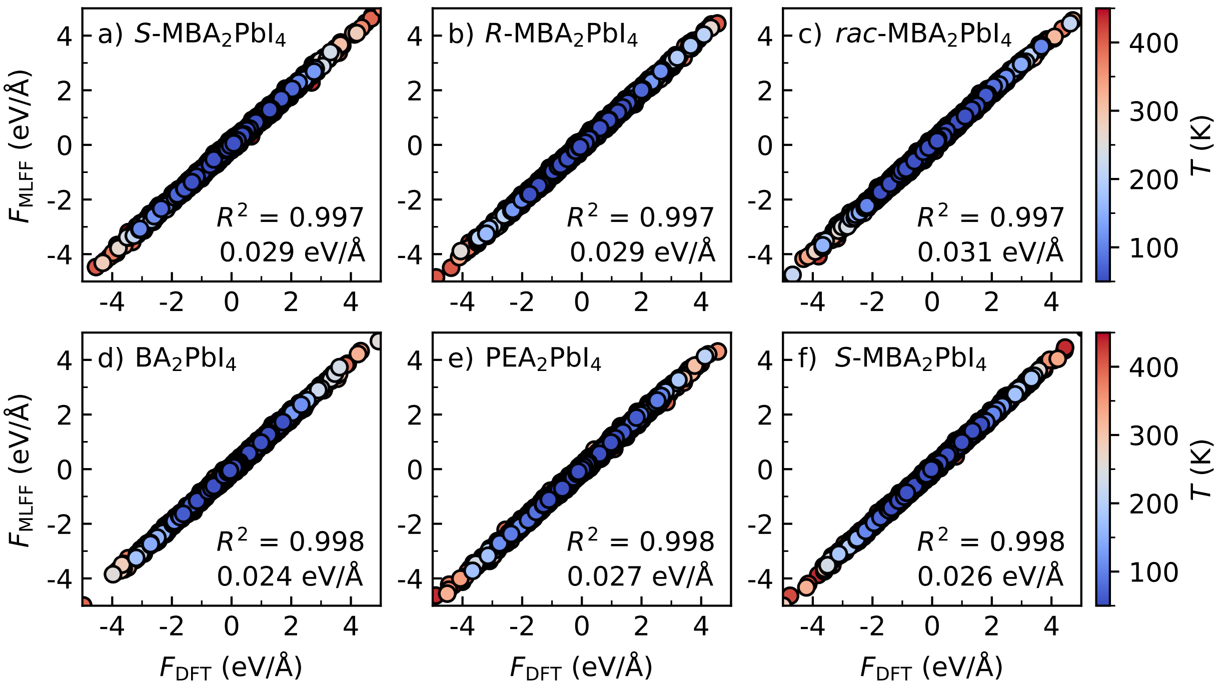

FIG. S5. Accuracy of machine-learning force fields (MLFFs) during short heating simulations from 50 K to 450 K. Model performance of MLFF trained on $(S\text{-MBA})_2PbI_4$ structures with the SCAN XC functional for (a) $(S\text{-MBA})_2PbI_4$, (b) $(rac\text{-MBA})_2PbI_4$ and (c) $(R\text{-MBA})_2PbI_4$ perovskite. Model performance of (d) BA_2PbI_4 and (e) PEA_2PbI_4 MLFF trained against SCAN XC functional and (e) $(S\text{-MBA})_2PbI_4$ MLFF trained against PBE+D3(BJ) XC functional. In each subfigure the coefficient of determination R^2 and mean absolute error MAE in eV Å^{-1} between the forces from the MLFF and DFT are given.

The model trained for (S-MBA)$_2$PbI$_4$ shows not only a high accuracy for the (S-MBA)$_2$PbI$_4$ perovskite structure it was trained against (Figure S5a), it can also accurately describe its enantiomer crystal (R-MBA)$_2$PbI$_4$ (Figure S5b). We attribute this finding to the invariance of the SOAP descriptor to reflections [18]. More notably, we observe that the MLFF trained on the chiral perovskite can also accurately describe the achiral (*rac*-MBA)$_2$PbI$_4$ (Figure S5c). Despite small structural differences between the chiral and achiral perovskite structures, the local environments of atoms are similar enough between the two to result in accurate MLFFs, only slightly increasing the MAE for the achiral perovskite. By training MLFFs against perovskites with different ligands (Figure S5d and Figure S5e) and using a different XC functional (Figure S5f) we show the accurate MLFFs can be obtained for a variety of perovskite systems.

C. Molecular dynamics simulations

The production molecular dynamics simulations made use of the same temperature and pressure control settings as used during the MLFF training, but we decreased the simulation time step to $\Delta t = 1\,\mathrm{fs}$ and set the hydrogen mass to $m_\mathrm{H} = 1\,\mathrm{u}$ to sample the system dynamics more accurately, analogous to when we tested the force field accuracy. At each temperature we started five independent constant temperature runs, the total length of each of these runs was 110 ps and the first 10 ps were discarded for system equilibration. The trajectories were sampled every 0.1 ps, resulting in a total of 5000 frames to analyze at each temperature. The structures used during the production runs consist of two inorganic layers and are expanded to similar dimensions in the lateral directions (i.e. a 3×3 supercell in the planar direction). Examples of such structures are shown in Figure S6 for (S-MBA)$_2$PbI$_4$ and (rac-MBA)$_2$PbI$_4$.

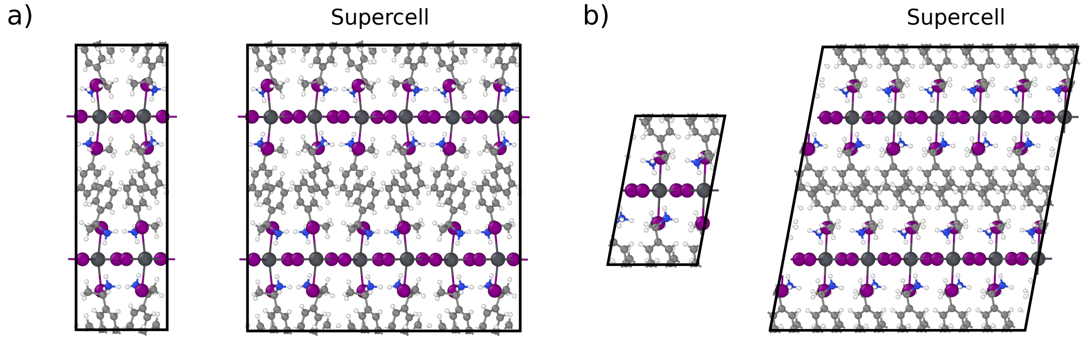

FIG. S6. Supercell geometries used in production runs. Unit cell and corresponding supercell for (a) (S-MBA)$_2$PbI$_4$ and (b) (rac-MBA)$_2$PbI$_4$.

Focusing on the MLFF trained for (S-MBA)$_2$PbI$_4$, we probe its ability to replicate experimentally determined perovskite geometries of (S-MBA)$_2$PbI$_4$ and (rac-MBA)$_2$PbI$_4$ perovskites [21]. Notably, the single model can accurately model the dimensions of both types of perovskite structures, as is shown in Figure S7. The MLFF simulations show a slight overprediction of the volumes of (S-MBA)$_2$PbI$_4$ (+2.2%) and (rac-MBA)$_2$PbI$_4$ (+1.5%) which appears to stem from the overprediction of the interlayer distances d in the structures.

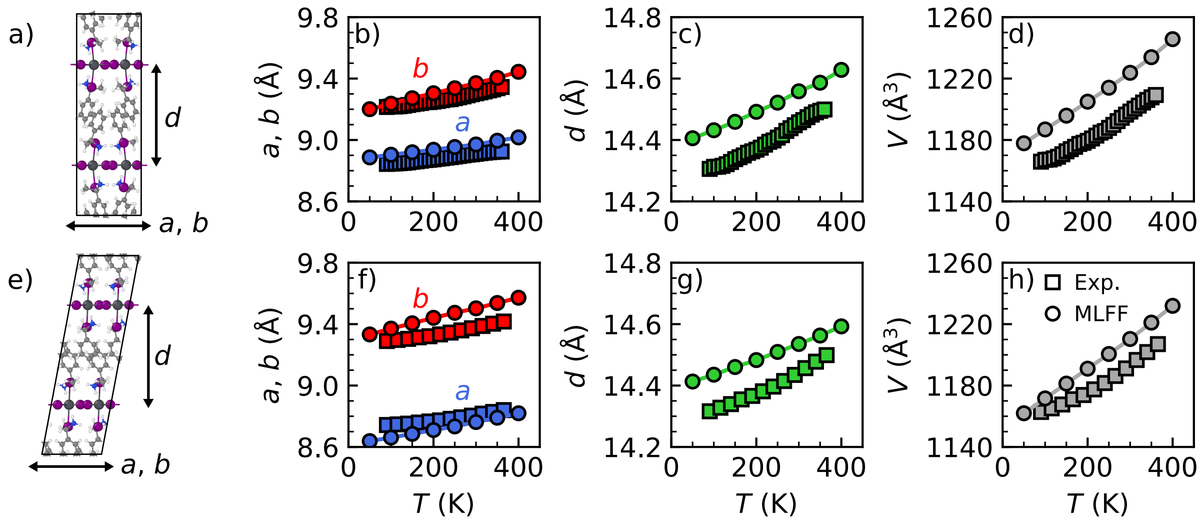

FIG. S7. Geometries of 2D perovskite structures from experiments and MLFF simulations. (a) (*S*-MBA)$_2$PbI$_4$ perovskite structure and its (b) lateral dimensions, (c) interlayer distances and (d) unit cell volumes. (e) (*rac*-MBA)$_2$PbI$_4$ perovskite structure and its (f) lateral dimensions, (g) interlayer distances and (h) unit cell volumes. Experimental data is shown in squares and the simulation data is shown in circles.

7. SI NOTE: DEGREE OF CHIRALITY

A. Uncertainty quantification of degree of chirality

To quantify the uncertainty in the degree of chirality of various descriptors, we investigate the spread in the values of χ^x for the five independent runs done at each temperature. As an example, the results of this uncertainty quantification (95% confidence interval) at 300 K for both (S-MBA)$_2$PbI$_4$ and (rac-MBA)$_2$PbI$_4$ are shown in Figure S8. The figure contains the data points extracted from each of the individual runs (white shapes) and the average and standard of this collection of data points (colored shapes with error bars). The results demonstrate a small spread in the degree of chirality for both perovskites. Notably, for (S-MBA)$_2$PbI$_4$ the error bars on the data are so small they are within the size of the shapes. For (rac-MBA)$_2$PbI$_4$ we find that the 95% confidence intervals contain the degree of chirality of zero. Altogether this indicates the use of the five independent simulation runs is sufficient to identify the difference between chiral and achiral perovskites.

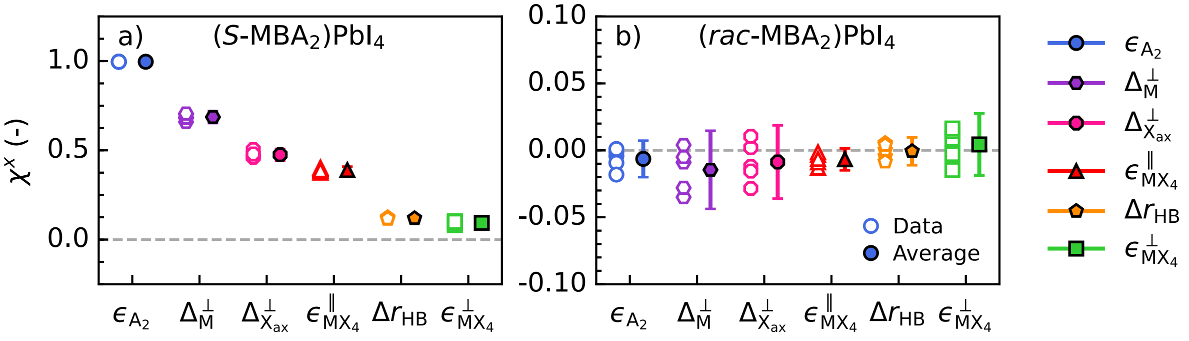

FIG. S8. Spread in the values of the structural descriptors determined at 300 K. Degree of chirality for various structural descriptors in (a) (S-MBA)$_2$PbI$_4$ and (b) (rac-MBA)$_2$PbI$_4$. Data points from the five different simulations are shown with the white shapes, the average of the five runs is shown in the colored shapes. All average values have their 95% confidence interval indicated along with them, when not shown the confidence interval is too narrow to see.

B. Temperature-dependence of chirality in (R-MBA)$_2$PbI$_4$

As done in the main text for (S-MBA)$_2$PbI$_4$ and (rac-MBA)$_2$PbI$_4$, the temperature dependence of the distributions of the structural descriptors and the degree of chirality can be computed for (R-MBA)$_2$PbI$_4$. The result of this analysis is shown in Figure S9. Due to the definition of the degree of chirality χ^x, we find negative values for the various structural descriptors in (R-MBA)$_2$PbI$_4$. We highlight the magnitudes of χ^x in (R-MBA)$_2$PbI$_4$ are the same as for (S-MBA)$_2$PbI$_4$, which follows from the mirror symmetry relation between the two enantiomer structures.

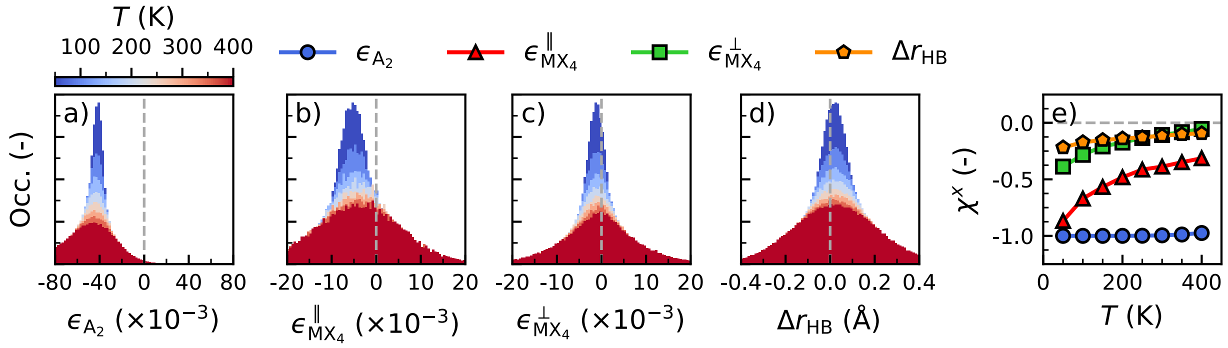

FIG. S9. Temperature dependence of the degree of chirality in (R-MBA)$_2$PbI$_4$. Temperature-dependent descriptor distributions for (a) cation chirality, (b) in-plane framework chirality, (c) out-of-plane framework chirality, and (d) hydrogen bond asymmetry. (e) Temperature-dependent degree of chirality.

C. Temperature-dependence of chirality in achiral perovskites

Analogous to MBA^+-based perovskites, we can determine the temperature dependence of the degree of chirality in achiral BA_2PbI_4 and PEA_2PbI_4. We subject both perovskite structures to a range of temperatures using the earlier-mentioned MLFFs trained for those perovskites. Note, due to structural phase transitions in BA_2PbI_4 at relatively low temperatures [22], we discarded the initial 60 ps of each individual simulation for BA_2PbI_4 data to allow for system equilibration. The results of these simulations are shown in Figure S10. For both perovskites, we observe a similar behavior as for achiral (rac-MBA)$_2$PbI$_4$, with the structures not showing any structural chirality for any of the investigated descriptors (i.e. ϵ_{A_2}, $\epsilon^{\parallel}_{MX_4}$, $\epsilon^{\perp}_{MX_4}$ and Δr_{HB}).

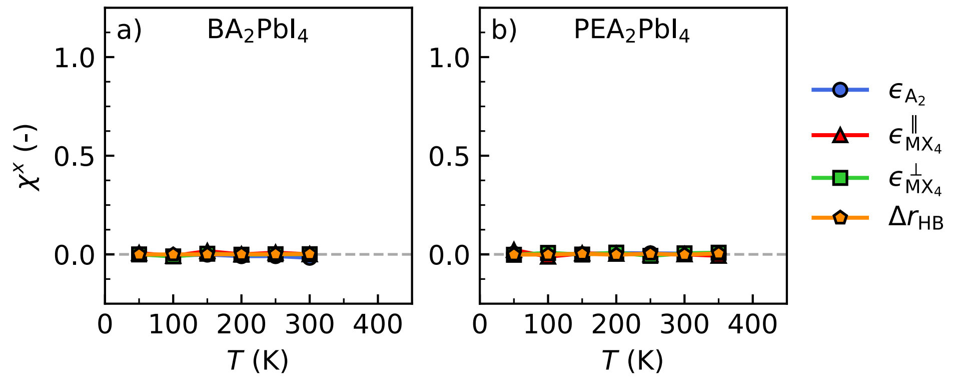

FIG. S10. Temperature dependence of the degree of chirality in achiral perovskites. Degree of chirality for (a) BA_2PbI_4 and (b) PEA_2PbI_4 perovskites.

D. Effects of exchange-correlation (XC) functional

All previous molecular dynamics results have been generated using MLFFs trained against data from DFT calculations using the SCAN XC functional. However, since we also trained a MLFF against DFT calculations with the PBE+D3(BJ) (Table S14), we can assess the influence that different XC functionals have on the degree of chirality. We show the temperature dependence of the degree of chirality for both SCAN and PBE+D3(BJ) in Figure S11 and provide some additional data in Table S15. Both functionals exhibit a decrease of the degree of chirality for the inorganic framework ($\epsilon^{\parallel}_{\mathrm{MX_4}}$ and $\epsilon^{\perp}_{\mathrm{MX_4}}$), but not for the arrangement of the organic cations ($\epsilon_{\mathrm{A_2}}$). At high temperatures (i.e. 400 K), irrespective of the functional the MLFF was trained against, a similar degree of chirality is found for all three descriptors for both MLFFs. This indicates that both models result in similar dynamics at such elevated temperatures. In contrast, some differences in the degree of chirality are found at low temperatures (i.e. 50 K), where PBE+D3(BJ)-based MLFF predicts a slightly lower degree of chirality for the in-plane frame work chirality ($\epsilon^{\parallel}_{\mathrm{MX_4}}$), but a somewhat larger degree of chirality for the out-of-plane framework chirality ($\epsilon^{\perp}_{\mathrm{MX_4}}$). We hypothesize these differences stem from the explicit inclusion of van der Waals forces in the PBE+D3(BJ) functional, which results in stronger interactions between the organic cations and inorganic framework, in turn leading to more severe out-of-plane distortions of the inorganic framework. Based on the more accurate unit cell volumes (SI Note 3) and previous benchmarks of XC functionals for hybrid halide perovskites [23, 24], we deem the dynamics from the SCAN-based MLFF to be more accurate.

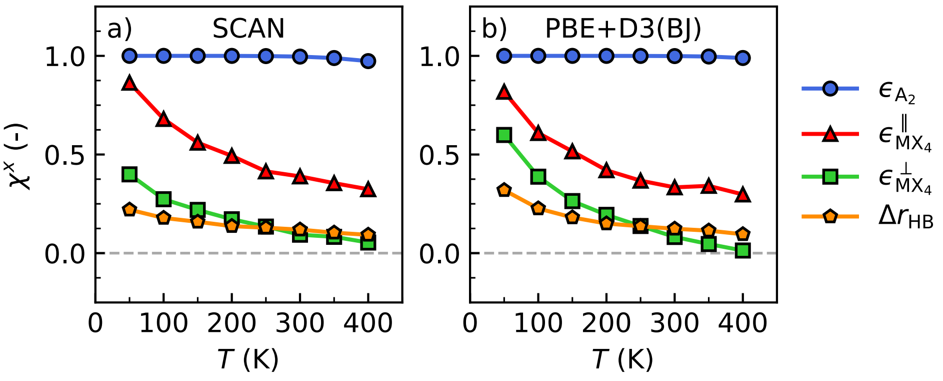

FIG. S11. Effects of different exchange-correlation (XC) functionals on the degree of chirality in descriptors. Temperature dependence of $\epsilon_{\mathrm{A_2}}$, $\epsilon^{\parallel}_{\mathrm{MX_4}}$ and $\epsilon^{\perp}_{\mathrm{MX_4}}$ using machine-learning force fields (MLFFs) trained against the (a) SCAN and (b) PBE+D3(BJ) XC functionals.

TABLE S15. Degrees of chirality for different XC functionals at different temperatures.

T (K)	XC functional	$\chi^{\epsilon_{A_2}}$ (-)	$\chi^{\epsilon^{\parallel}_{MX_4}}$ (-)	$\chi^{\epsilon^{\perp}_{MX_4}}$ (-)
50	SCAN	1.000	0.864	0.399
	PBE+D3(BJ)	1.000	0.818	0.599
400	SCAN	0.973	0.324	0.054
	PBE+D3(BJ)	0.989	0.298	0.013

8. SI NOTE: CHIRALITY TRANSFER

A. Effects of exchange-correlation (XC) functional

To assess the influence of the XC functional on the dynamics of the NH_3^+ group, we compare the orientational autocorrelation functions in Figure S12. The simulations with both the SCAN (Figure S12a) and PBE+D3(BJ) (Figure S12b) functionals, exhibit a very similar time and temperature dependence. Interestingly, the typical rotation time of the NH_3^+ group $\tau_{NH_3^+}$ for both functionals at 400 K is similar, at 6.0 ps and 6.9 ps for the MLFF trained using SCAN and PBE+D3(BJ), respectively. We connect this similarity in the dynamics at elevated temperatures to the above-mentioned similarity in the degree of chirality between the two MLFFs.

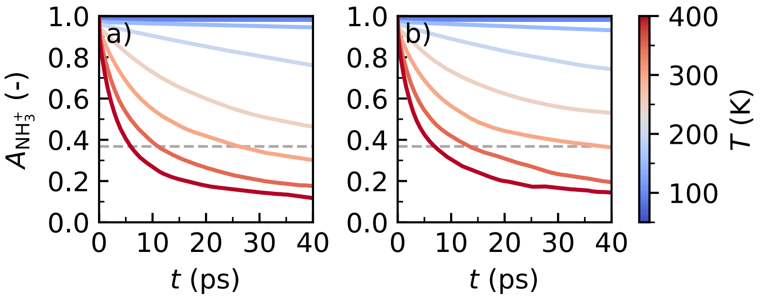

FIG. S12. Dependency of cation headgroup orientational autocorrelation function $A_{NH_3^+}$ on exchange-correlation (XC) functional for the (a) SCAN and (b) PBE+D3(BJ) XC functionals. The typical rotation time for the cation headgroups in $(S\text{-MBA})_2PbI_4$ at 400 K is 6.0 ps and 6.9 ps for the MLFFs trained against SCAN and PBE+D3(BJ), respectively.

B. Cation reorientations

In addition to determining the orientational autocorrelation of the N − H bonds in the NH_3^+ groups, we can also employ the autocorrelation function to assess the persistence of the orientation of the organic cations. To do so, we use orientation vectors that span the length ($\mathbf{r}_l$) and width ($\mathbf{r}_w$) of the MBA^+ cations, as shown in Figure S13a. The vectors can be defined using the atomic fingerprints from SI Note 2 as shown in Table S16. The orientational autocorrelation functions of these vectors (Figure S13b-e) level off at values close to 1. This indicates a highly persistent orientation of the MBA^+ cations in both (S-MBA)$_2$PbI$_4$ (Figure S13b-c) and (rac-MBA)$_2$PbI$_4$ (Figure S13d-e), where the organic cations do not exhibit any rotations.

TABLE S16. Atom fingerprints used to determine the orientation of the MBA^+ cation.

Vector	Element 1	Atom fingerprint 1	Element 2	Atom fingerprint 2
$\mathbf{r}_l$	C	H32C16N1	C	H52C18N5
$\mathbf{r}_w$	C	H46C16N4	C	H46C16N4

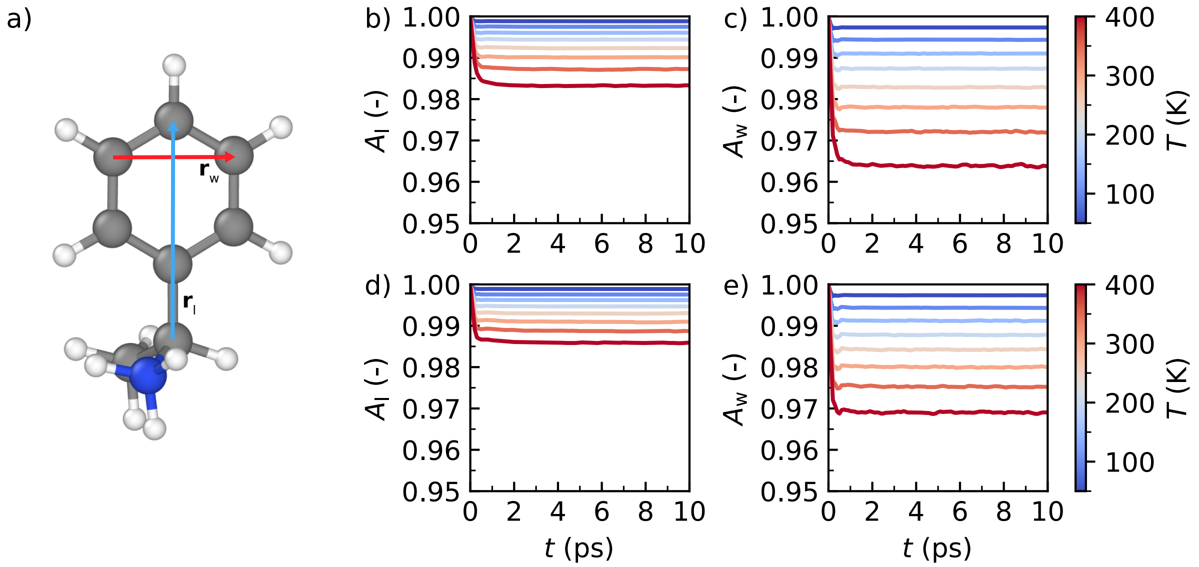

FIG. S13. Orientational correlation function for organic cations. (a) The length $\mathbf{r}_\mathrm{l}$ and width $\mathbf{r}_\mathrm{w}$ vectors used to define the cation orientation. The orientational autocorrelation of the (b) length and (c) width vectors in (S-MBA)$_2$PbI$_4$ and (d) length and (e) width vectors in (rac-MBA)$_2$PbI$_4$.

9. SI NOTE: DISTORTIONS AND HYDROGEN BONDS IN PEROVSKITES

To compare the degree with which perovskite structures are distorted, we compare the values of several structural descriptors for 2D perovskite structures with the MBA^+, $1NEA^+$ and $2NEA^+$ cation. The results of this analysis are shown in Table S17. Distortions of the inorganic framework are captured by the bond angle variance σ^2 and the bond length variance Δd. For all investigated cations, the chiral structures (i.e. *S*-/*R*-) have larger distortions in the inorganic framework than the achiral racemic counterparts, which is in line with earlier work in literature [2, 14]. We focus ourselves on the nature of the hydrogen bonds between the organic cations and inorganic framework in the various perovskites, by investigating the hydrogen bond lengths in the inorganic cages. To do so, we report the hydrogen bond length of the strongly ($\bar{r}_{\mathrm{HB}}^{\mathrm{strong}}$) and weakly ($\bar{r}_{\mathrm{HB}}^{\mathrm{weak}}$) bound cation to the cages as well as the average hydrogen bond length ($\bar{r}_{\mathrm{HB}}$) of these cations. Here we find that for all cations the chiral structures (*S*-/*R*-) bind the cations more weakly, as illustrated by the longer hydrogen bond lengths, when compared to the racemic perovskite structures. We hypothesize the larger framework distortions and more weakly bound organic cations come about due to the breaking of symmetry that results from the chiral cations, which also distorts the framework and weakens the hydrogen bonds.

TABLE S17. Information of inorganic framework distortions and hydrogen bond strength for various 2D perovskite structures with chiral organic cations. The data is from structures optimized using the SCAN XC functional.

Structure	σ^2 ($^{\circ 2}$)	Δd ($\times 10^{-5}$)	$\bar{r}_{\mathrm{HB}}^{\mathrm{strong}}$ (Å)	$\bar{r}_{\mathrm{HB}}^{\mathrm{weak}}$ (Å)	$\bar{r}_{\mathrm{HB}}$ (Å)
(*S*-MBA)$_2$PbI$_4$	20.17	29.38	2.605	2.640	2.623
(*R*-MBA)$_2$PbI$_4$	20.17	29.38	2.605	2.640	2.623
(*rac*-MBA)$_2$PbI$_4$	14.04	18.19	2.595	2.595	2.595
(*S*-1NEA)$_2$PbBr$_4$	42.48	75.48	2.364	2.408	2.386
(*R*-1NEA)$_2$PbBr$_4$	43.21	70.85	2.358	2.416	2.387
(*rac*-1NEA)$_2$PbBr$_4$	22.58	20.88	2.345	2.345	2.345
(*S*-2NEA)$_2$PbBr$_4$	48.15	47.24	2.325	2.423	2.374
(*R*-2NEA)$_2$PbBr$_4$	49.53	48.14	2.319	2.427	2.373
(*rac*-2NEA)$_2$PbBr$_4$	23.43	8.97	2.315	2.315	2.315

[1] K. Robinson, G. V. Gibbs, and P. H. Ribbe, Science **172**, 567 (1971).

[2] M. K. Jana, R. Song, H. Liu, D. R. Khanal, S. M. Janke, R. Zhao, C. Liu, Z. Valy Vardeny, V. Blum, and D. B. Mitzi, Nat. Commun. **11**, 4699 (2020).

[3] M. K. Jana, R. Song, Y. Xie, R. Zhao, P. C. Sercel, V. Blum, and D. B. Mitzi, Nat. Commun. **12**, 4982 (2021).

[4] S. Apergi, G. Brocks, and S. Tao, J. Phys. Chem. Lett. **14**, 11565 (2023).

[5] G. Kresse and J. Hafner, Phys. Rev. B **49**, 14251 (1994).

[6] G. Kresse and J. Furthmüller, Comput. Mater. Sci. **6**, 15 (1996).

[7] G. Kresse and J. Furthmüller, Phys. Rev. B **54**, 11169 (1996).

[8] J. Sun, A. Ruzsinszky, and J. P. Perdew, Phys. Rev. Lett. **115**, 036402 (2015).

[9] J. P. Perdew, K. Burke, and M. Ernzerhof, Phys. Rev. Lett. **77**, 3865 (1996).

[10] S. Grimme, S. Ehrlich, and L. Goerigk, J. Comput. Chem. **32**, 1456 (2011).

[11] G. Kresse and D. Joubert, Phys. Rev. B **59**, 1758 (1999).

[12] H. J. Monkhorst and J. D. Pack, Phys. Rev. B **13**, 5188 (1976).

[13] Y. Dang, X. Liu, Y. Sun, J. Song, W. Hu, and X. Tao, J. Phys. Chem. Lett. **11**, 1689 (2020).

[14] J. Son, S. Ma, Y.-K. Jung, J. Tan, G. Jang, H. Lee, C. U. Lee, J. Lee, S. Moon, W. Jeong, A. Walsh, and J. Moon, Nat. Commun. **14**, 3124 (2023).

[15] K.-z. Du, Q. Tu, X. Zhang, Q. Han, J. Liu, S. Zauscher, and D. B. Mitzi, Inorg. Chem. **56**, 9291 (2017).

[16] M. Menahem, Z. Dai, S. Aharon, R. Sharma, M. Asher, Y. Diskin-Posner, R. Korobko, A. M. Rappe, and O. Yaffe, ACS Nano **15**, 10153 (2021).

[17] R. Jinnouchi, F. Karsai, and G. Kresse, Phys. Rev. B **100**, 014105 (2019).

[18] A. P. Bartók, R. Kondor, and G. Csányi, Phys. Rev. B **87**, 184115 (2013).

[19] M. Parrinello and A. Rahman, Phys. Rev. Lett. **45**, 1196 (1980).

[20] M. Parrinello and A. Rahman, J. Appl. Phys. **52**, 7182 (1981).

[21] I. Dursun, M. W. Terban, Y. Xu, X. Zhang, S. Ghose, M. Li, P. Sarker, H. Li, M. Cotlet, T. Wei, D. Lu, and Q. Yu, J. Phys. Chem. C **127**, 15423 (2023).

[22] R.-I. Biega, M. Bokdam, K. Herrmann, J. Mohanraj, D. Skrybeck, M. Thelakkat, M. Retsch, and L. Leppert, J. Phys. Chem. C **127**, 9183 (2023).

[23] M. Bokdam, J. Lahnsteiner, B. Ramberger, T. Schäfer, and G. Kresse, Phys. Rev. Lett. **119**, 145501 (2017).

[24] J. Lahnsteiner, G. Kresse, J. Heinen, and M. Bokdam, Phys. Rev. Mater. **2**, 073604 (2018).
